# Brown Macroalgal Polyphenols and Oxidative Stress: A Systematic Review and Meta-Analysis of In Vivo Evidence in Vertebrate Models

**DOI:** 10.3390/antiox15080961

**Published:** 2026-07-31

**Authors:** Dolores Cejalvo-Lapeña, Desiré Sánchez-Bonet, Samanta García-Oms, Mariola Belda-Antolí, José Miguel Lloris-Cejalvo, Carolina Padrón-Sanz

**Affiliations:** 1 Centro de Investigación Traslacional San Alberto Magno (CITSAM), Catholic University of Valencia San Vicente Mártir, 46001 Valencia, Spain; dolores.cejalvo@ucv.es (D.C.-L.); mariola.belda@ucv.es (M.B.-A.); 2 Doctoral School, Catholic University of Valencia San Vicente Mártir, 46001 Valencia, Spain; desanbo88@mail.ucv.es (D.S.-B.); samanta.garcia@ucv.es (S.G.-O.); jm.lloris@ucv.es (J.M.L.-C.)

**Keywords:** brown algae, polyphenols, animal model, antioxidant, phlorotannins, oxidative stress

## Abstract

Brown macroalgal polyphenols, particularly phlorotannins, have demonstrated antioxidant and anti-inflammatory bioactivity, but in vivo evidence remains heterogeneous across organisms, stress models, and preparations. This systematic review and meta-analysis synthesized in vivo evidence on the effects of brown macroalgal polyphenols on oxidative stress-related biomarkers. PubMed, Cochrane, Scopus, and Web of Science were searched. Of 415 records identified, 22 in vivo studies involving zebrafish embryos, mice, and rats met the inclusion criteria. Interventions included extracts, fractions, and purified compounds. Random-effects meta-analyses were performed for prespecified outcomes, with subgroup analyses by compound, macroalgal species, and stressor/model. In zebrafish, brown macroalgal polyphenols reduced ROS generation, lipid peroxidation, and nitric oxide production. In rodents, they increased catalase activity and serum superoxide dismutase, and reduced hepatic malondialdehyde and TBARS, whereas glutathione peroxidase showed no consistent pooled effect. Risk-of-bias assessment using SYRCLE indicated predominantly unclear reporting for randomization, allocation concealment, and blinding, while attrition and reporting domains were mostly low-risk. Overall, brown macroalgal polyphenols show promising antioxidant effects in vivo, but substantial heterogeneity and incomplete methodological reporting limit certainty and preclude direct extrapolation to humans.

## 1. Introduction

Oxidative stress and inflammation are tightly coupled biological processes that contribute to tissue injury in a wide spectrum of acute and chronic conditions. Therefore, they are widely used to evaluate candidate protective compounds [1]. Marine-derived polyphenols have garnered increasing interest due to their structural diversity and broad range of bioactivities [2]. Phlorotannins are a distinctive class of phenolic compounds found almost exclusively in brown macroalgae (Phaeophyceae) [3]. Structurally, they are polymers of phloroglucinol linked through C–C and/or C–O–C bonds, generating multiple subclasses with a broad molecular weight range that may influence their pharmacokinetics and biological activities [4,5]. Depending on the nature of the inter-unit linkages, they are commonly classified as fucols (aryl–aryl bonds), phlorethols (aryl–ether bonds), fucophlorethols (mixed aryl–aryl and aryl–ether bonds), eckols (dibenzodioxin-type structures), and carmalols (dibenzodioxin-containing derivatives) [5]. The representative compounds included in this review, such as phloroglucinol, dieckol, eckol, triphlorethol, phlorofucofuroeckol-A, octaphlorethol A, and diphlorethohydroxycarmalol, belong mainly to the eckol, phlorethol, and fucophlorethol subclasses. Both the structural class and degree of polymerization are key determinants of physicochemical properties, including solubility, bioavailability, and biological activity, and may therefore contribute to heterogeneity between studies.

Preclinical studies have repeatedly linked brown-algal phlorotannin, particularly eckol derivatives and higher-order oligomers, to antioxidant and anti-inflammatory effects in vivo, including attenuation of reactive oxygen species (ROS) generation, lipid peroxidation, inflammatory mediators, apoptosis, and histopathological damage in organ-specific toxicity and disease models [6,7]. Mechanistically, many studies have focused on key redox- and inflammation-regulating pathways, such as Nrf2/HO-1 signaling and NF-κB inhibition, although these effects may vary depending on the compound composition, dosage, and exposure conditions [4,7]. The translational appeal extends beyond nutraceutical development to topical and cosmeceutical concepts, where antioxidant/anti-inflammatory activities are frequently positioned as mechanistic rationales for skin-protective or anti-aging applications [8].

However, the in vivo evidence base remains fragmented, with substantial heterogeneity in algae sources, extraction methods, experimental models, and outcomes, limiting direct comparability and complicating inferences about which preparations, species, or stressors yield the most consistent effects. Zebrafish embryos (*Danio rerio*) are commonly used for rapid in vivo screening of oxidative injury using ROS-sensitive fluorescent probes after pro-oxidant challenges, whereas rodent experiments typically quantify enzymatic antioxidants and lipid peroxidation biomarkers across tissues [9]. In addition, incomplete reporting of randomization and blinding can undermine confidence in preclinical effect estimates, making structured appraisal essential. SYRCLE’s risk-of-bias tool provides a domain-based framework tailored to animal intervention studies [10].

Although numerous reviews have described the chemistry, structural diversity, and biological activities of marine polyphenols and phlorotannins [2,3,4,5,11], or focused on specific applications such as anticancer activity [7], cosmeceuticals [8,12,13], anti-inflammatory effects [14,15], cardiovascular health [16], and nutraceuticals [17], most are narrative, combining in vitro and in vivo evidence without quantitative synthesis, or are limited to particular compounds or applications. To our knowledge, this is the first systematic review and meta-analysis to quantitatively evaluate the in vivo effects of brown macroalgal polyphenols/phlorotannins on oxidative stress biomarkers in vertebrate models using standardized effect sizes, subgroup analyses, the PRISMA methodology, and structured risk-of-bias assessment.

A meaningful evaluation of the biological activity of brown macroalgal polyphenols/phlorotannins requires evidence obtained under physiologically integrated conditions, where absorption, distribution, metabolism, and excretion (ADME), tissue pharmacokinetics, and systemic regulatory pathways remain intact [9,18,19]. Consequently, vertebrate in vivo models constitute the most appropriate experimental framework for assessing their antioxidant efficacy.

Moreover, the limited bioavailability and extensive metabolism of high-molecular-weight phlorotannins reduce the predictive value of in vitro antioxidant assays [11,20,21].

Consequently, vertebrate in vivo models provide the most informative framework for evaluating antioxidant efficacy under physiologically integrated conditions and for generating robust preclinical evidence with greater translational relevance to human health [22].

Accordingly, this systematic review and meta-analysis aimed to comprehensively synthesize vertebrate in vivo evidence on the effects of brown macroalgal polyphenols/phlorotannins on oxidative stress-related biomarkers, to quantify these effects through meta-analytic pooling where data permit using standardized mean differences under a random-effects framework, to explore heterogeneity through prespecified subgroup analyses by compound/extract class, macroalgal species, stressor/model, and sample tissue, to evaluate internal validity using the SYRCLE risk-of-bias tool, and thereby to provide, to our knowledge, the first PRISMA-compliant quantitative synthesis of the field in order to inform the design of future primary studies and to support the prioritization of candidate preparations for translational nutraceutical and cosmeceutical development.

## 2. Materials and Methods

### 2.1. Protocol Registration

This systematic review and meta-analysis were conducted in accordance with the Preferred Reporting Items for Systematic Reviews and Meta-Analyses (PRISMA) statement and the Cochrane Handbook for Systematic Reviews and Meta-Analysis [23,24]. Our systematic review protocol was registered in OSF (registration ID: 10.17605/OSF.IO/PXT93).

### 2.2. Data Sources and Search Strategy

A comprehensive, filter-free electronic search was performed in PubMed (MEDLINE), Scopus, Web of Science, and the Cochrane Central Register of Controlled Trials (CENTRAL) from inception to January 2026. Search terms were constructed to capture brown macroalgae and common genera (e.g., *Ecklonia*, *Sargassum*, *Ishige*, *Fucus*), polyphenols/phlorotannins and key compounds (e.g., dieckol, phloroglucinol, phlorofucofuroeckol-A), and in vivo experimental models and oxidative/inflammatory injury paradigms including UV-B, AAPH, LPS, high glucose, ethanol, H_2_O_2_, and ionizing radiation. Database-specific syntax and the full search strategy are provided in the Supplementary Materials (Supplementary Table S1). Reference lists of eligible full texts were also screened to identify additional relevant studies.

### 2.3. Eligibility Criteria

We included in vivo experimental studies evaluating brown macroalgal polyphenols/phlorotannins as crude extracts, enriched fractions, or purified compounds, administered to zebrafish and/or mammalian models represented by mice or rats, with a comparator group of vehicle or untreated control. Eligible studies were required to report at least one quantifiable oxidative stress, antioxidant enzyme, inflammatory, or tissue injury endpoint relevant to the review question, including outcomes such as ROS generation, lipid peroxidation, MDA, TBARS, NO, and enzymatic antioxidants including CAT, GPx and SOD. Both preventive (pre-exposure or co-exposure) and therapeutic or post-insult designs were eligible.

We excluded in vitro studies, ex vivo or cell culture experiments, human clinical studies, reviews, editorials, letters, conference abstracts without full data, book chapters, and non-English articles. When multiple publications reported overlapping datasets, the most complete report or the one with the most relevant extractable outcome data was retained.

### 2.4. Study Selection

All retrieved records were imported into Rayyan for de-duplication and screening. After removal of duplicates, titles and abstracts were screened for relevance, followed by full-text assessment of potentially eligible reports against predefined criteria. Study selection was performed in duplicate, and disagreements were resolved by discussion and consensus; when required, a senior reviewer was consulted.

### 2.5. Data Extraction

Data were extracted independently in duplicate using a piloted standardized extraction sheet, with the collection of study characteristics including first author, year, country, study design, animal species and strain, sample size, sex when available, and follow-up duration when applicable, alongside model characteristics such as the experimental insult or stressor and the relevant disease or toxicity model. Intervention details were recorded for each study, covering the algae species or source material, extract type, compound identity, route of administration, dosing regimen, timing distinguishing preventive from therapeutic use, and comparator information. The outcomes of interest included continuous data for oxidative stress markers and antioxidant enzyme activities with means and standard deviations or convertible statistics, as well as the tissue or sample type and timing of outcome assessment.

When numerical results were reported only in figures, the values were extracted using a standardized digital plot-reading approach. If dispersion measures were not directly reported, conversions were performed using established methods (e.g., SE to SD) [24] when sufficient information was available. Any discrepancies in extraction were reconciled by consensus.

### 2.6. Risk of Bias Assessment

Risk of bias for animal studies was assessed using the SYRCLE Risk of Bias tool, evaluating domains aligned with selection bias including sequence generation, baseline characteristics and allocation concealment, performance bias assessing random housing and blinding of caregivers/investigators, then detection bias including random outcome assessment and blinding of outcome assessment, along with attrition bias (incomplete outcome data), reporting bias (selective outcome reporting), and other sources of bias [10]. Each domain was judged as low, high, or unclear risk. Assessments were performed independently by two reviewers and disagreements were resolved through discussion, with adjudication by a senior reviewer if necessary.

### 2.7. Statistical Analysis

Quantitative synthesis was undertaken when ≥2 studies reported the same outcome with extractable data. Conversely, studies that met the inclusion criteria but did not provide extractable quantitative data for any prespecified outcome (e.g., outcomes reported only descriptively, only in graphical form without extractable values, or only as non-convertible summary statistics) were retained in the systematic review as a qualitative ‘review subset’, but they did not contribute to the pooled effect estimates. Similarly, studies whose outcomes could not be pooled because fewer than two studies contributed to the same outcome were also retained in the review subset. For continuous outcomes measured on different scales across studies, effect sizes were summarized using standardized mean differences (SMDs) with 95% confidence intervals (CI). Specifically, the SMD was computed as Hedges’ g, a bias-corrected version of Cohen’s d that adjusts for small sample sizes, with 95% confidence intervals derived from the standard error of the pooled estimate [24]. Between-study variance (τ^2^) was estimated using the restricted maximum likelihood (REML) method, as recommended for continuous outcomes in the Cochrane Handbook for Systematic Reviews of Interventions [24]. When outcomes were reported on comparable scales within a subgroup, the same SMD framework was retained to ensure consistency across pooled analyses. Meta-analyses were performed using a random-effects model to account for expected between-study heterogeneity arising from differences in species, stressor paradigms, intervention formulations, dosing, and sampling matrices.

Statistical heterogeneity was quantified using I^2^ and Cochran’s χ^2^ test. Prespecified subgroup analyses were conducted to explore heterogeneity by compound/extract class (purified phlorotannins vs. polyphenol-rich fractions/extracts), algal species (e.g., *Ecklonia cava* vs. other brown algae), stressor/model type (e.g., UV-B, high glucose, AAPH, ethanol, LPS, H_2_O_2_, radiation), and sample type/tissue for rodent antioxidant enzyme outcomes (serum, liver, kidney, muscle, erythrocytes). Tests for subgroup differences were reported where applicable.

Sensitivity analyses included leave-one-out analyses to assess the influence of individual studies on pooled estimates. Small-study effects/publication bias were evaluated visually using funnel plots and statistically using Egger’s regression test when the number of studies permitted meaningful assessment. All tests were two-sided, and *p* < 0.05 was considered statistically significant. All analyses were performed in R 4.3.3 using the meta package.

## 3. Results

### 3.1. Literature Search

**Figure 1:**
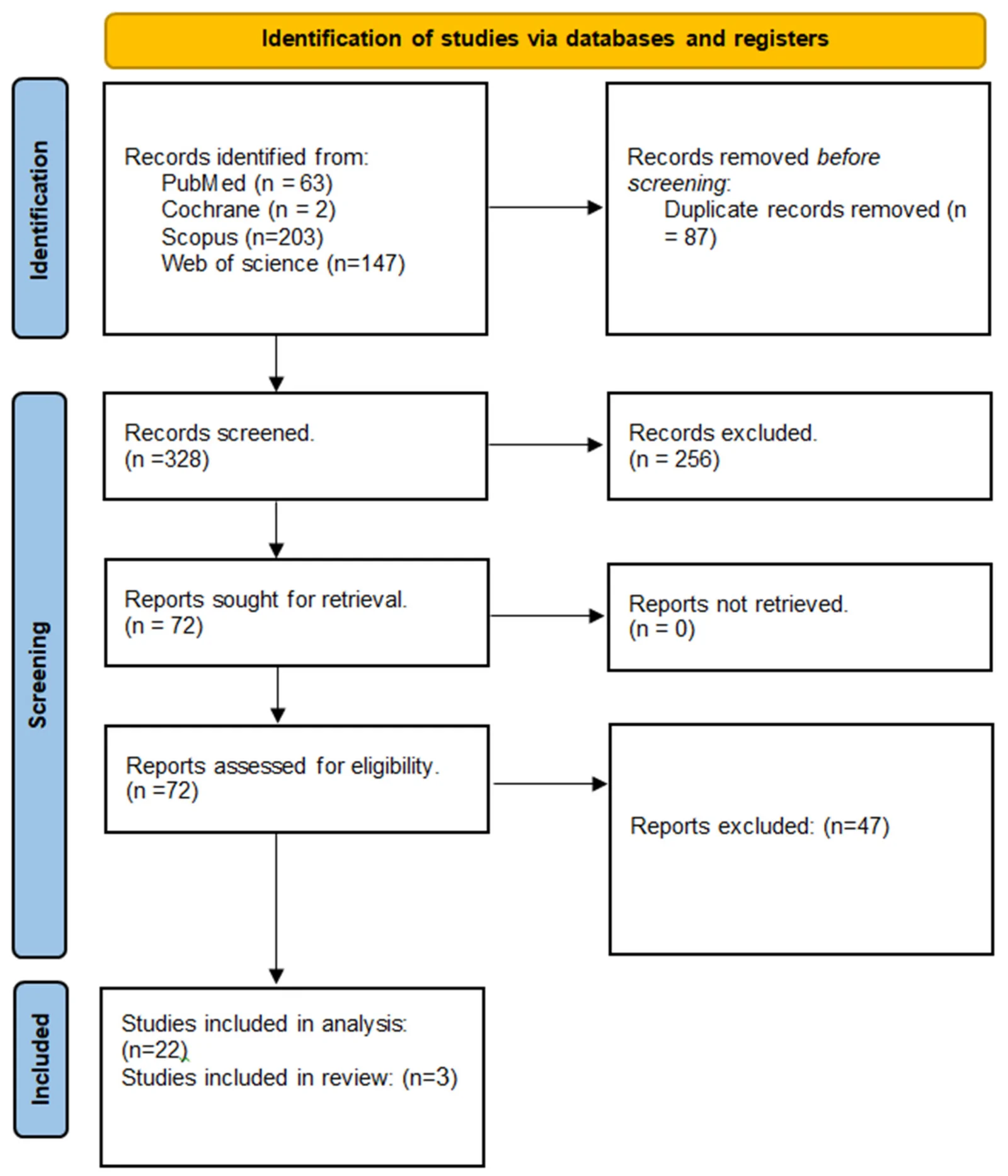
PRISMA 2020 flow diagram showing the identification, screening, eligibility, and inclusion of studies. Twenty-five studies fulfilled the inclusion criteria; 22 were included in the quantitative synthesis (meta-analysis), whereas 3 were retained only for the qualitative synthesis.

A total of 415 records were initially identified from databases (PubMed = 63, Cochrane = 2, Scopus = 203, Web of Science = 147). After removing 87 duplicate records, 328 records remained and were screened. During screening, 256 records were excluded, leaving 72 reports for full-text retrieval and eligibility assessment. All 72 reports were successfully retrieved, but 47 reports were excluded after full-text evaluation. Ultimately, 25 studies met the inclusion criteria and were included in this systematic review [6,25,26,27,28,29,30,31,32,33,34,35,36,37,38,39,40,41,42,43,44,45,46,47,48]. Of these, 22 studies contributed extractable quantitative data to one or more meta-analyses (i.e., reported group means with a dispersion measure, or convertible statistics, for at least one pre-specified oxidative stress or antioxidant endpoint) and were included in the final quantitative analysis [6,26,27,28,29,30,31,32,35,36,37,38,39,40,41,42,43,44,45,46,47,48], whereas the remaining three studies were retained only in the qualitative review subset, because the reported outcomes were not directly comparable with the pre-specified oxidative stress biomarkers of the meta-analysis, and the available statistics could not be reliably converted to the required format [25,33,34]. Accordingly, these three studies are summarized in the descriptive results but do not contribute to the pooled effect estimates. The PRISMA flowchart is shown in Figure 1.

### 3.2. Characteristics of Included Studies

Table 1 and Supplementary Table S2 summarize the included in vivo studies, mostly from South Korea/Republic of Korea, with additional studies from China, India, and Spain, evaluating brown macroalgal polyphenols/phlorotannins, predominantly from *Ecklonia cava*, but also *Sargassum* sp., *Ishige* sp., *Fucus vesiculosus*, and processing by-products, in zebrafish embryos, mice, and rats. Most experiments used oxidative/inflammatory stress models such as UV-B, AAPH, high glucose, LPS, ethanol, H_2_O_2_, and ionizing radiation, with additional disease/toxicity models including type 2 diabetes (db/db mice), CCl_4_ liver injury, KBrO_3_ nephrotoxicity, NDEA hepatocarcinogenesis, glucocorticoid-induced osteoporosis, sepsis, and radiation dermatitis.

The interventions ranged from crude extracts/fractions to purified compounds, such as dieckol, phloroglucinol, octaphlorethol A, phlorofucofuroeckol-A, and DPHC, delivered mainly by oral gavage/diet or waterborne immersion (zebrafish), with one topical formulation used. The dosing varied widely (µg/mL–µM in zebrafish; mg/kg–g/kg in rodents) and was predominantly preventive, with fewer therapeutic designs reported. Overall, the studies consistently reported antioxidant, anti-inflammatory, and tissue-protective effects, typically reductions in ROS, lipid peroxidation, inflammatory mediators, apoptosis, and organ injury markers, sometimes linked to NF-κB and Nrf2/HO-1 pathways, supporting potential nutraceutical/cosmeceutical applications, while also highlighting heterogeneity in models, formulations, and endpoints that limit direct cross-study comparability and human extrapolation.

### 3.3. Risk of Bias Assessment

**Figure 2:**
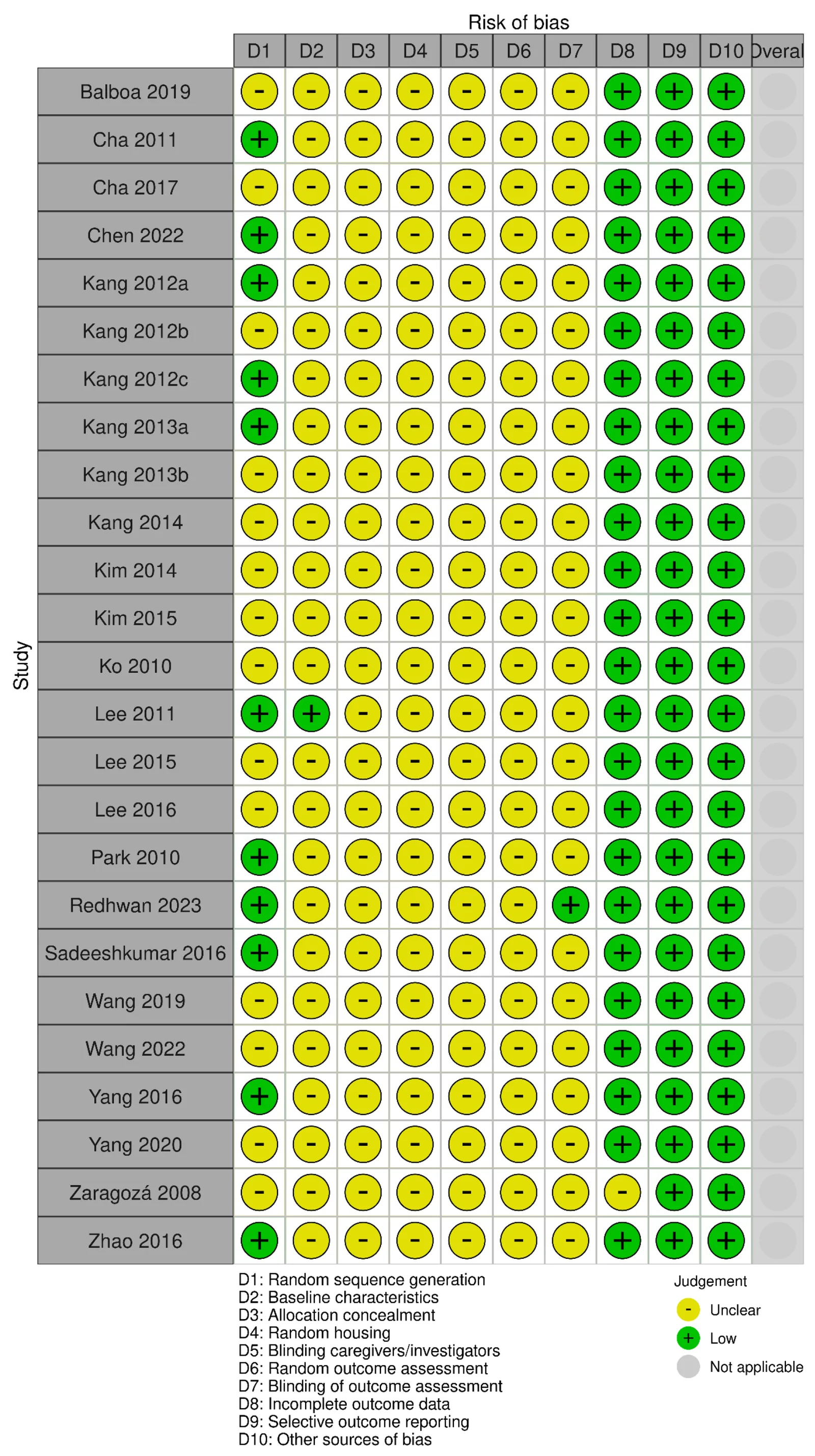
Risk of bias assessment using SYRCLE tool [6,25,26,27,28,29,30,31,32,33,34,35,36,37,38,39,40,41,42,43,44,45,46,47,48].

Across studies, the SYRCLE domains that map to selection and performance/detection bias safeguards are mostly “unclear” (yellow), especially allocation concealment, random housing, blinding of caregivers/investigators, random outcome assessment, and blinding of outcome assessors, which typically reflects insufficient reporting rather than confirmed high risk. In contrast, attrition bias (incomplete outcome data) and reporting/other bias are predominantly low-risk, suggesting limited loss-to-follow-up/missing data and no strong signals of selective reporting. Overall, the SYRCLE assessment indicates that confidence is constrained mainly by poorly described randomization/blinding procedures, while downstream outcome handling/reporting appears comparatively stronger. Summary of risk of bias assessment is illustrated in Figure 2.

### 3.4. ROS Generation for Zebrafish Models

**Figure 3:**
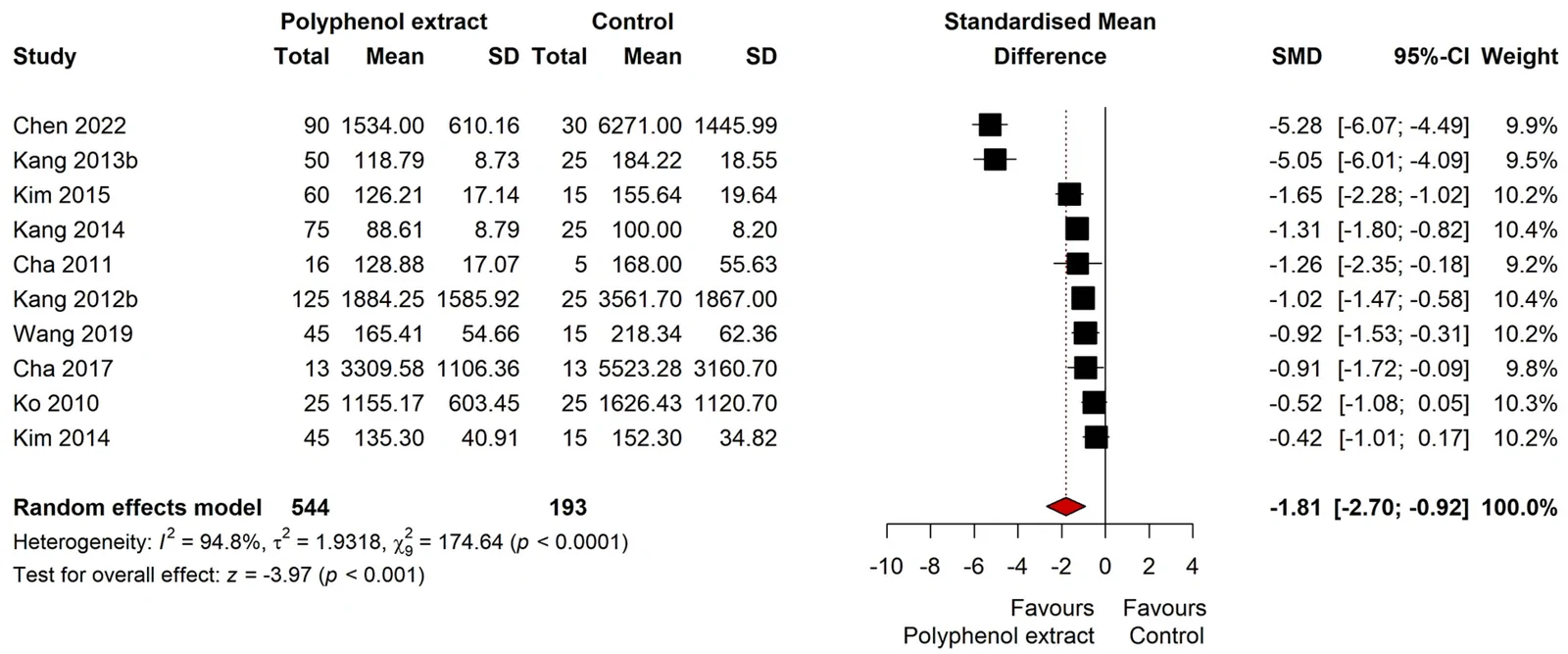
Forest plot of random-effects meta-analysis of ROS generation in zebrafish models comparing polyphenol/phlorotannin treatment vs. control [29,36,37,38,39,40,43,45,46,47].

Across 10 studies, the random-effects meta-analysis shows that polyphenol extracts significantly reduced the outcome compared with controls (SMD = −1.81, 95% CI −2.70 to −0.92, *p* < 0.001) (Figure 3).

**Figure 4:**
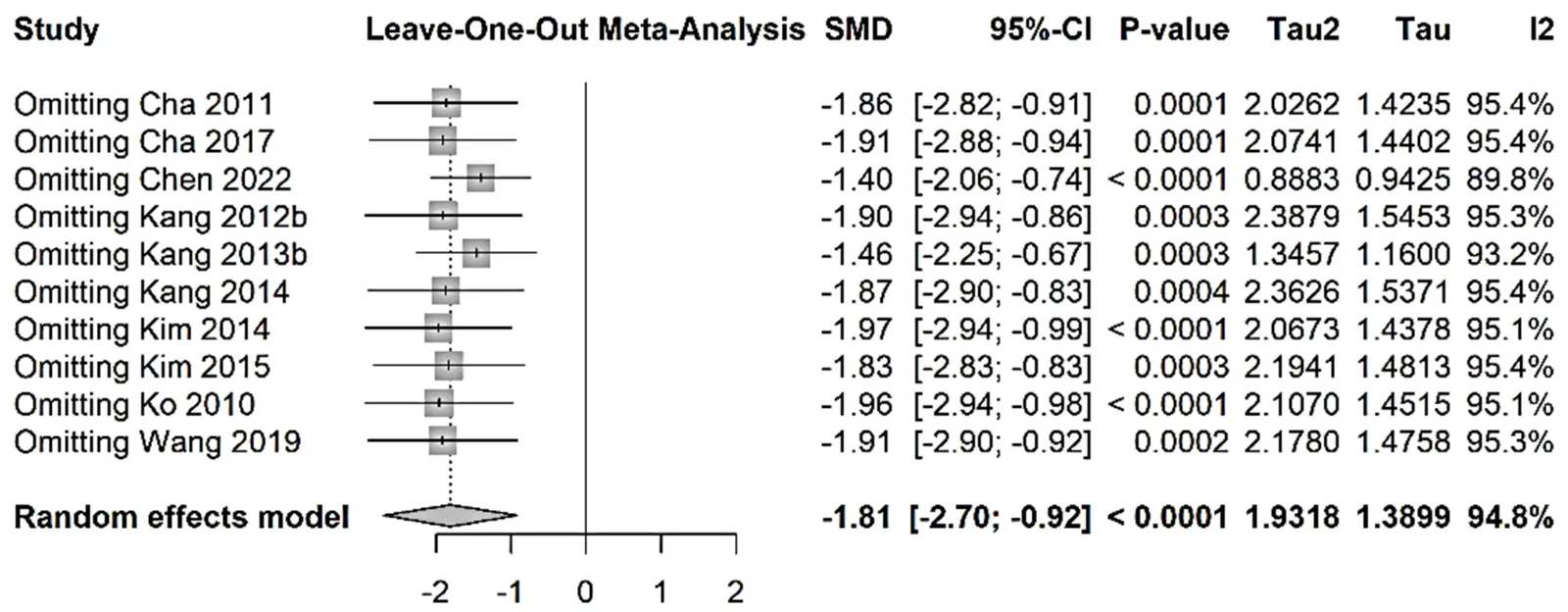
Leave-one-out sensitivity analysis for the zebrafish ROS meta-analysis [29,36,37,38,39,40,43,45,46,47].

Nevertheless, heterogeneity is extremely high (I^2^ = 94.8%, χ^2^ *p* < 0.0001). The leave-one-out analysis shows the pooled effect remains consistently negative and statistically significant regardless of which study is removed (SMDs roughly between −1.4 to −2.0), suggesting the overall finding is not driven by a single study, although heterogeneity remains high (Figure 4).

The funnel plot does not show strong evidence of small-study/publication bias by Egger’s test (*p* = 0.1155), but visual asymmetry is difficult to judge with this small number of studies and marked heterogeneity (Supplementary Figure S1).

**Figure 5:**
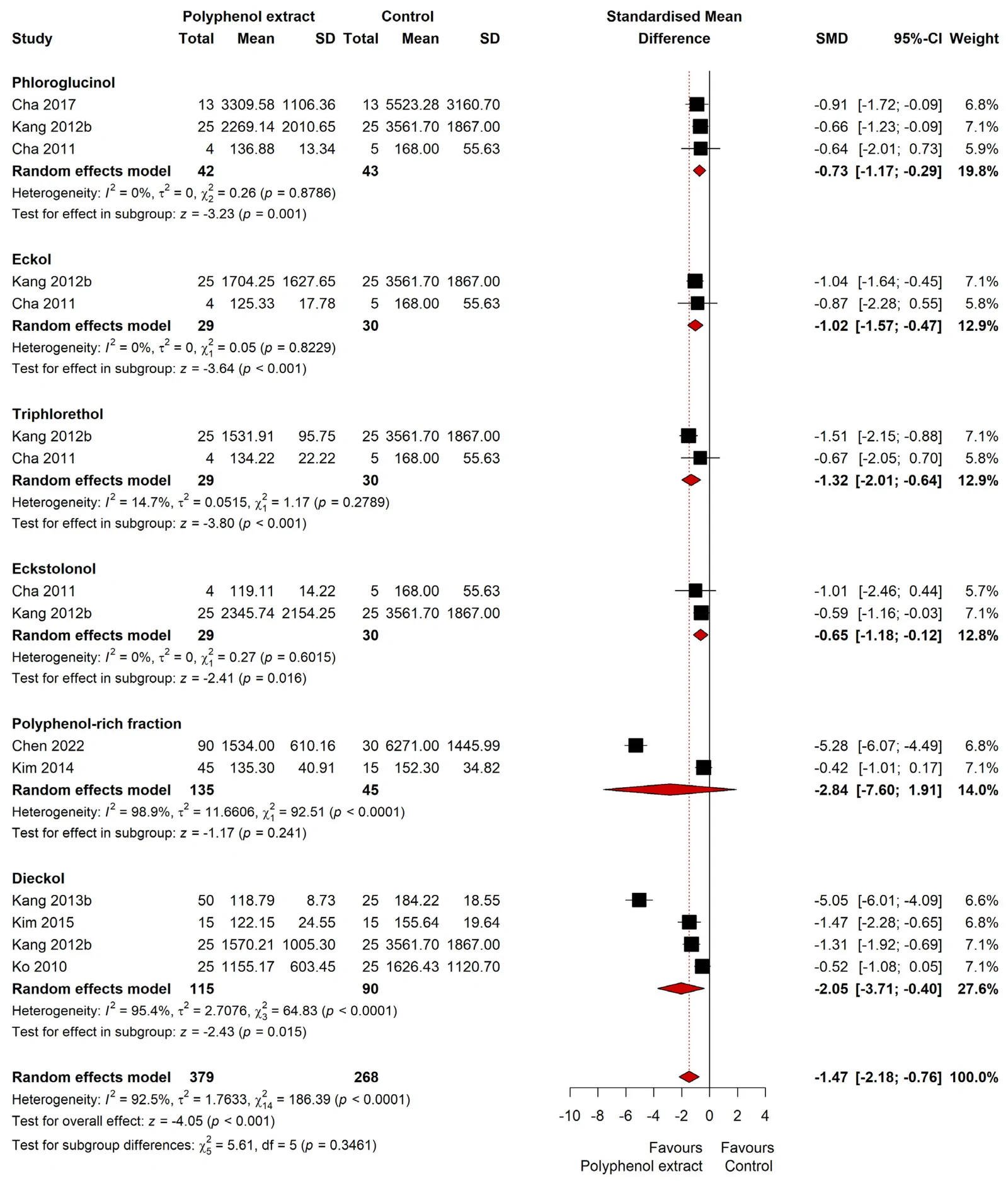
Subgroup meta-analysis of zebrafish ROS by intervention type/compound [36,37,38,40,43,45,46,47].

Subgroup analysis shows that most individual phlorotannins (phloroglucinol, eckol, triphlorethol, eckstolonol) have significant protective effects with low heterogeneity within subgroups (I^2^ = 0–15%). In contrast, polyphenol-rich fractions and especially dieckol show larger but highly heterogeneous effects, and overall, the pooled random-effects estimate still favors polyphenol extract (SMD = −1.47, 95% CI −2.18 to −0.76), with no significant subgroup differences (*p* = 0.35) (Figure 5).

When stratified by brown macroalgae species, the effects remained in favor of polyphenol extract, with the evidence base dominated by *Ecklonia cava* (SMD = −1.51, 95% CI −2.41 to −0.60) but with high heterogeneity (I^2^ = 92%). Single-study subgroups for *Sargassum thunbergii*, *Ishige foliacea*, and *Ishige okamurae* also showed negative SMDs, and the overall pooled effect was unchanged (SMD = −1.81, 95% CI −2.70 to −0.92); importantly, subgroup differences were significant (*p* < 0.0001), suggesting that species-level variation contributes to the between-study heterogeneity (Supplementary Figure S2).

Stratification by stressor/model showed that the overall effect still favored polyphenol extract (SMD = −1.81, 95% CI −2.70 to −0.92), but the effects varied markedly across exposures (subgroup differences *p* < 0.0001). The high-glucose subgroup was consistently protective with no heterogeneity (SMD = −1.44, 95% CI −1.82 to −1.05; I^2^ = 0%), whereas the UV-B subgroup showed a large but imprecise and borderline pooled effect (SMD = −1.99, 95% CI −4.09 to 0.11; *p* = 0.063) with very high heterogeneity (I^2^ = 97%). Other stressors (H_2_O_2_ liver injury, AAPH, ethanol, and LPS) were represented by single studies, each generally favoring the extract, except for LPS, which was not clearly significant, limiting firm within-stressor conclusions (Supplementary Figure S3).

### 3.5. CAT Production

**Figure 6:**
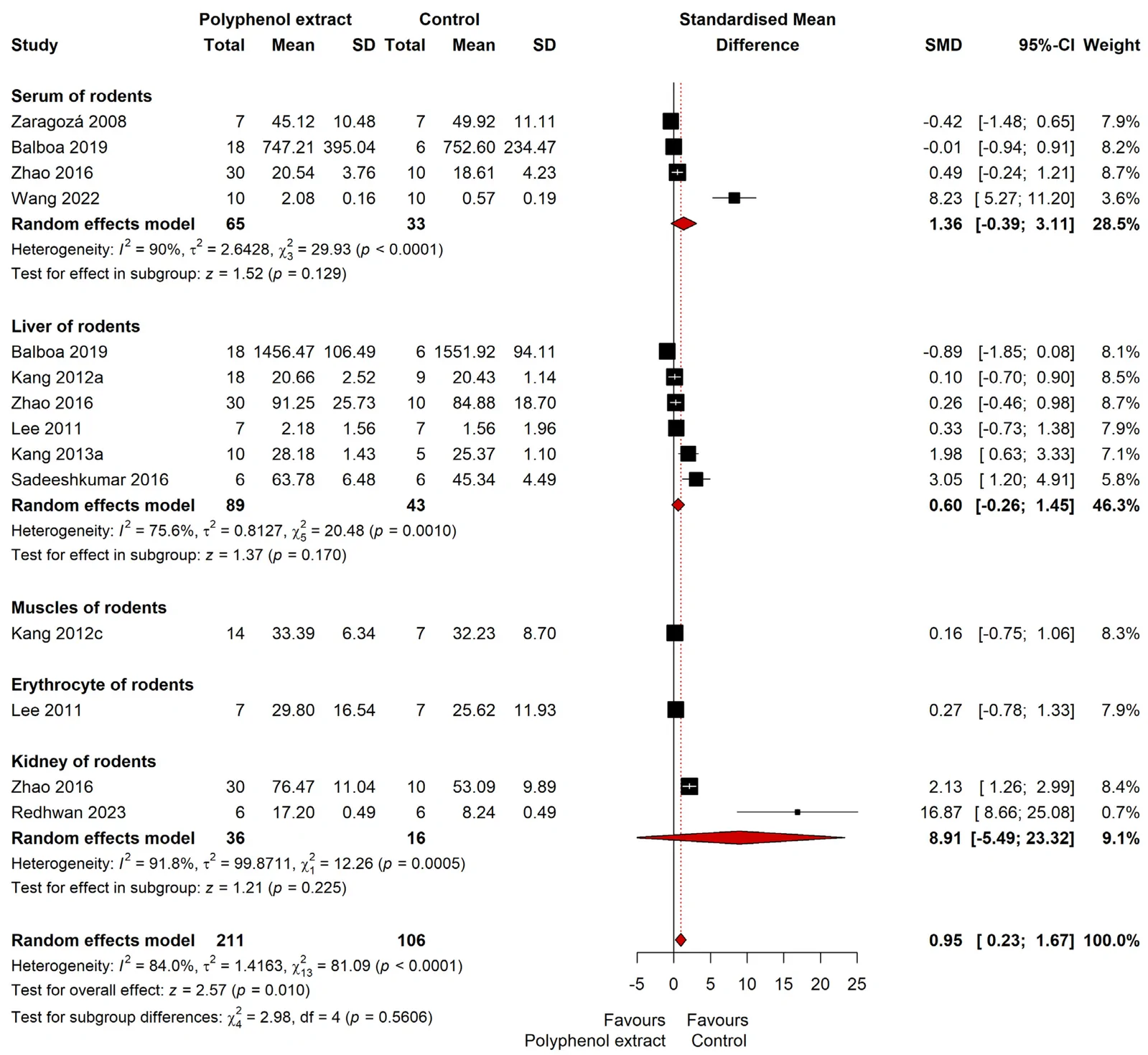
Forest plot of catalase (CAT) activity in rodent models, subgrouped by sample type [26,27,28,30,31,35,41,42,44,48].

Under the CAT production outcome, subgrouping by sample type in rodents shows a modest overall increase with polyphenol extract versus control (SMD = 0.95, 95% CI 0.23–1.67; *p* = 0.01) despite considerable heterogeneity (I^2^ = 84%). Within subgroups, serum (SMD 1.36, 95% CI −0.39 to 3.11), liver (SMD 0.60, 95% CI −0.26 to 1.45), and kidney (SMD 8.91, 95% CI −5.49 to 23.32) each have wide, non-significant CIs with high within-subgroup heterogeneity (serum/kidney), while muscle and erythrocyte are single, non-significant estimates. The test for subgroup differences is not significant (*p* = 0.56) (Figure 6).

Building on the rodent sample-type analysis, ordering the same CAT production data by species (rats vs. mice) shows that most rat subgroups remain non-significant with very high heterogeneity, while kidney in rats is driven by a single study with a very large positive effect. In mice, the pooled estimate for liver is modest and non-significant (SMD 0.51, 95% CI −0.15 to 1.17) with moderate heterogeneity, whereas single mouse samples suggest clearer increases for kidney (SMD 2.13, 95% CI 1.26 to 2.99) but not for muscle, erythrocyte, or serum. Overall, subgroup differences are statistically significant (*p* = 0.0002) (Supplementary Figure S4).

### 3.6. GPx Production

**Figure 7:**
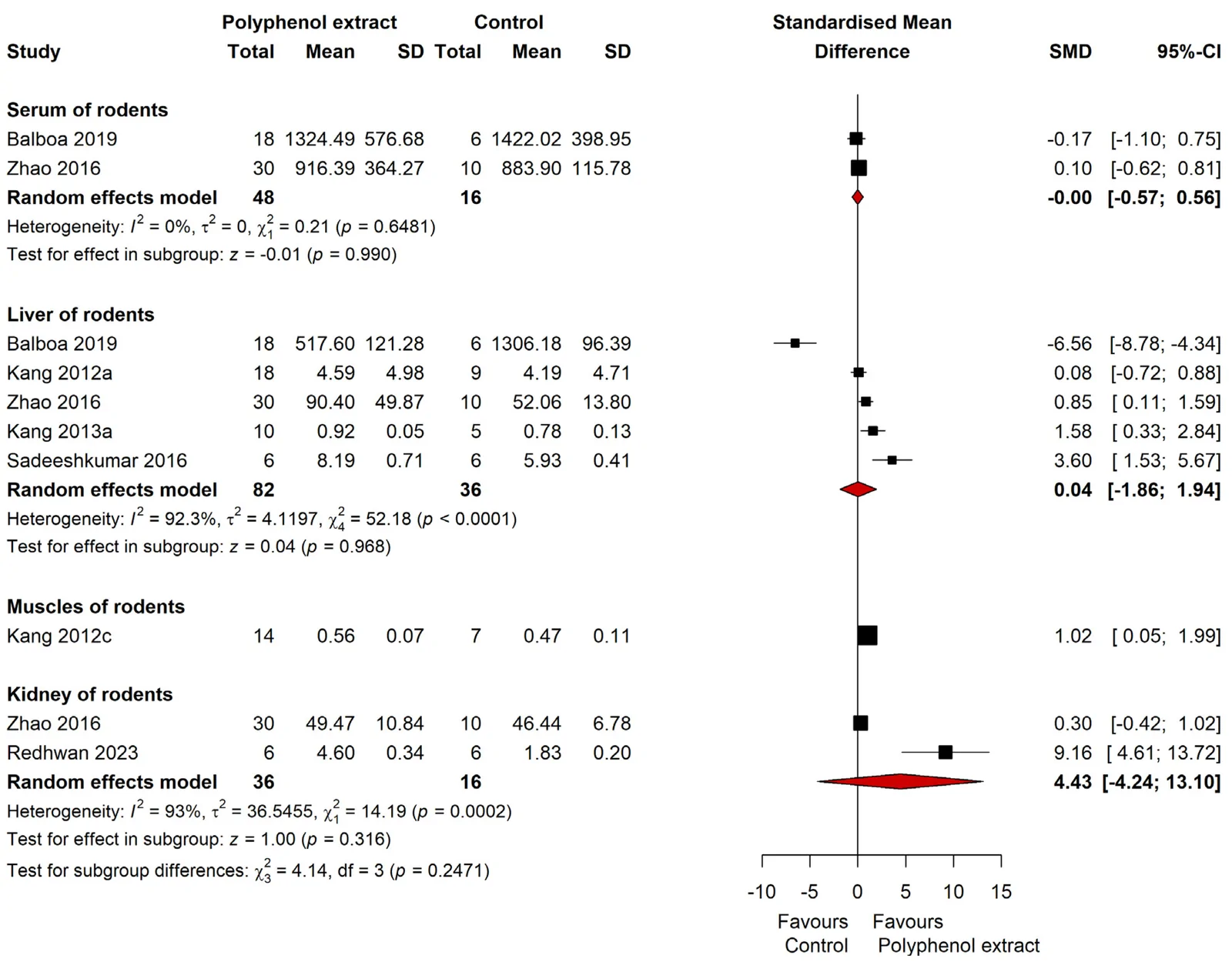
Forest plot of glutathione peroxidase (GPx) activity in rodent models, subgrouped by sample type [26,30,31,41,42,44,48].

For GPx production in rodents, subgrouping by sample type showed no clear overall effect of polyphenol extract across tissues, as serum was essentially null with no heterogeneity (SMD 0.00, 95% CI −0.57 to 0.56), while the liver (SMD 0.04, 95% CI −1.86 to 1.94) and kidney (SMD 4.43, 95% CI −4.24 to 13.10) had very wide, non-significant CIs with high heterogeneity (I^2^ = 92–93%). The only tissue showing a statistically significant increase was muscle (single study, SMD 1.02, 95% CI 0.05–1.99). Overall, the subgroup differences were not significant (*p* = 0.25) (Figure 7). Extending the GPx sample-type analysis to species-stratified subgroups (rats vs. mice) showed mixed and largely inconclusive effects, as liver estimates in rats were highly heterogeneous and the pooled liver effect was non-significant (SMD −1.47, 95% CI −11.42 to 8.48), while serum was null and the kidney was represented by a single large positive study. In mice, the liver showed a borderline increase (SMD 0.74, 95% CI −0.03 to 1.50; *p* = 0.061), the muscle showed a significant increase (single study SMD 1.02, 95% CI 0.05–1.99), and the serum/kidney were non-significant single-study estimates. Overall, the subgroup differences were significant (*p* = 0.0044) (Supplementary Figure S5).

### 3.7. MDA Production

**Figure 8:**
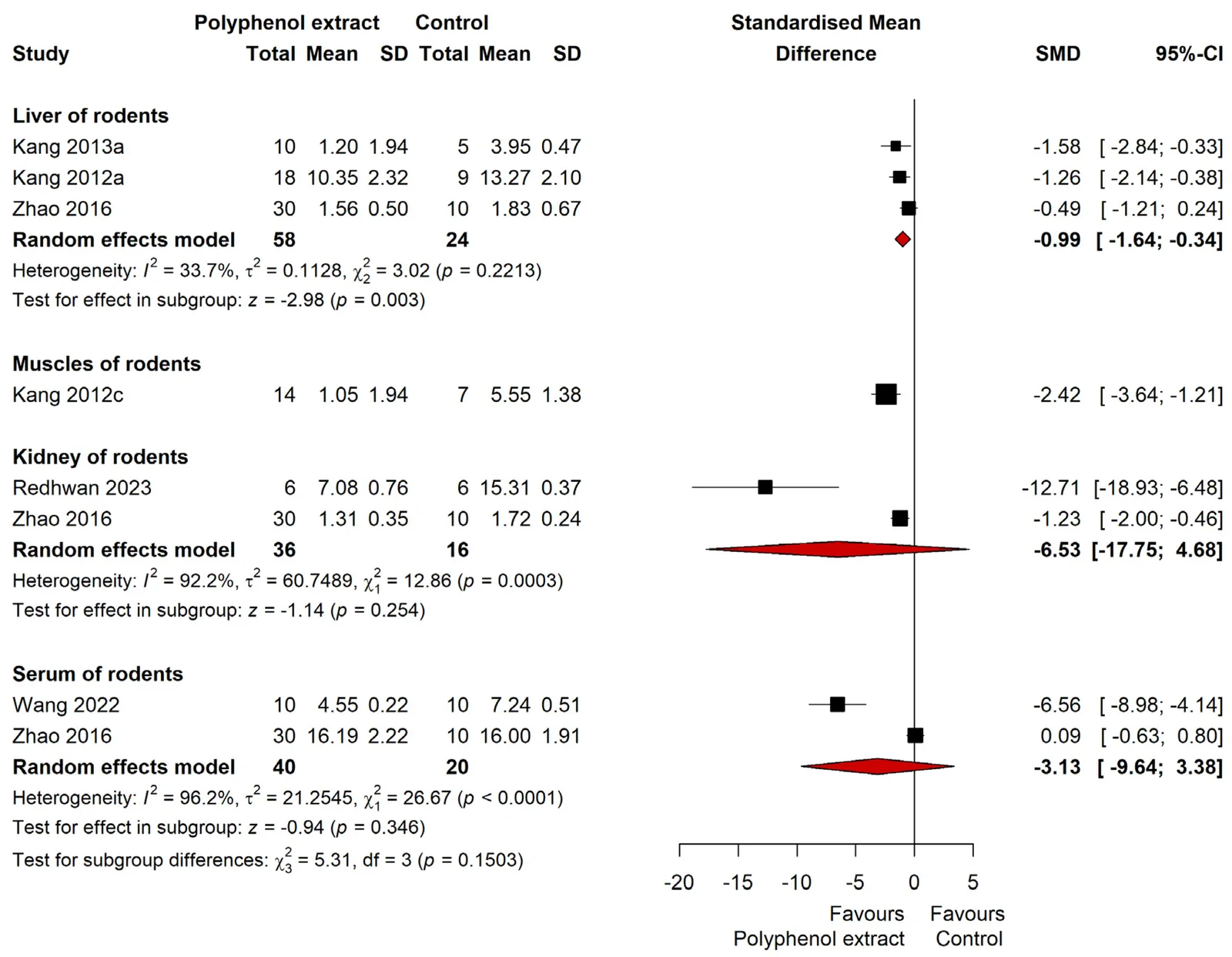
Forest plot of malondialdehyde (MDA) levels in rodent models, subgrouped by sample type [26,28,31,41,42,44].

For MDA production, subgrouping by rodent sample type shows effects largely favouring polyphenol extract, with the most consistent signal in liver (SMD = −0.99, 95% CI −1.64 to −0.34, *p* = 0.003; I^2^ = 33.7%). Muscle also shows a marked reduction (single study SMD = −2.42, 95% CI −3.64 to −1.21). In contrast, kidney and serum subgroups have very high heterogeneity (I^2^ = 92–96%) with wide, non-significant pooled estimates (kidney SMD = −6.53, 95% CI −17.75 to 4.68; serum SMD = −3.13, 95% CI −9.64 to 3.38). The test for subgroup differences is not significant (*p* = 0.15) (Figure 8).

When the MDA results are ordered by species (rats vs. mice), the direction generally still favours polyphenol extract, but the pattern differs across strata as the rat evidence is sparse and tissue-specific with large reductions in kidney and serum, whereas the mouse data provide the more stable estimate, showing a significant reduction in liver (SMD −0.99, 95% CI −1.64 to −0.34) and a strong reduction in muscle (single study SMD −2.42, 95% CI −3.64 to −1.21), with kidney also significant (single study SMD −1.23, 95% CI −2.00 to −0.46) but serum null. Overall, subgroup differences are significant (*p* < 0.0001) (Supplementary Figure S6).

### 3.8. SOD Production

**Figure 9:**
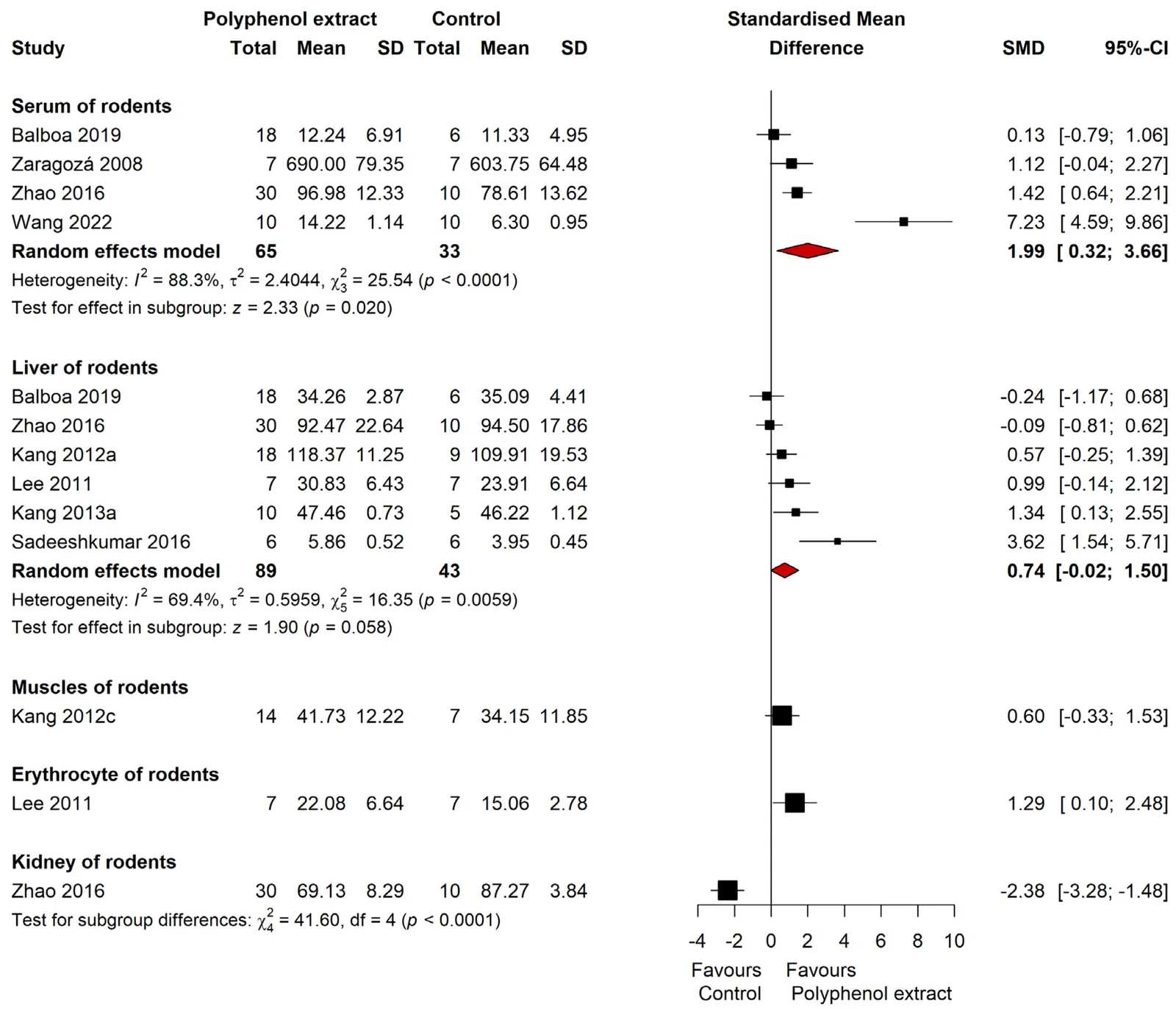
Forest plot of superoxide dismutase (SOD) activity in rodent models, subgrouped by sample type [26,27,28,30,35,41,42,44,48].

For SOD production in rodents, effects varied by tissue, with serum showing a significant increase with polyphenol extract (SMD 1.99, 95% CI 0.32–3.66; I^2^ = 88%), while the liver showed a borderline, non-significant increase (SMD 0.74, 95% CI −0.02 to 1.50; I^2^ = 69%). Single-study estimates suggested increases in erythrocytes (SMD 1.29, 95% CI 0.10–2.48) but not clearly in muscle (SMD 0.60, 95% CI −0.33 to 1.53), whereas the kidney showed a significant effect in the opposite direction (single study SMD −2.38, 95% CI −3.28 to −1.48). Overall, the subgroup differences were significant (*p* < 0.0001) (Figure 9). When SOD production was stratified by species, the apparent increases in SOD were mainly observed in the serum and liver, but the pooled effects were borderline/non-significant in both rat serum (SMD 2.49, 95% CI −0.29 to 5.26; *p* = 0.08) and rat liver (SMD 1.57, 95% CI −2.21 to 5.36; *p* = 0.42) with very high heterogeneity. In mice, the liver showed a small, borderline increase (SMD 0.57, 95% CI −0.04 to 1.19; *p* = 0.068), while single studies indicated higher SOD in serum (SMD 1.42, 95% CI 0.64–2.21) and erythrocytes (SMD 1.29, 95% CI 0.10–2.48), but a significant effect in the opposite direction in the kidney (SMD −2.38, 95% CI −3.28 to −1.48). Overall, subgroup differences remain significant (*p* < 0.0001) (Supplementary Figure S7).

### 3.9. Lipid Peroxidation, NO Production and TBARS Production

**Figure 10:**
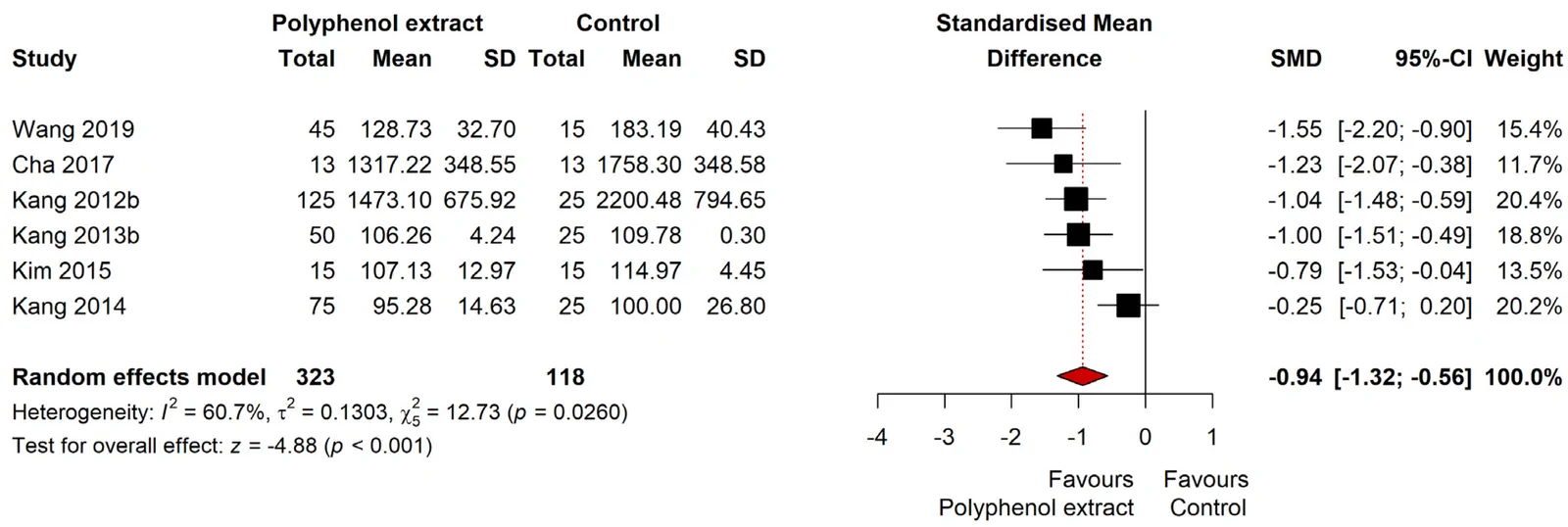
Forest plot of random-effects meta-analysis of lipid peroxidation (DPPP oxide) in zebrafish models comparing polyphenol/phlorotannin treatment vs. control [29,37,39,40,43,47].

In zebrafish models, polyphenol treatment significantly reduced lipid peroxidation (DPPP oxide) compared with controls under a random-effects model (SMD −0.94, 95% CI −1.32 to −0.56, *p* < 0.001). Most individual studies favor the extract, with only one estimate crossing the null, and heterogeneity is moderate (I^2^ = 60.7%) (Figure 10).

**Figure 11:**
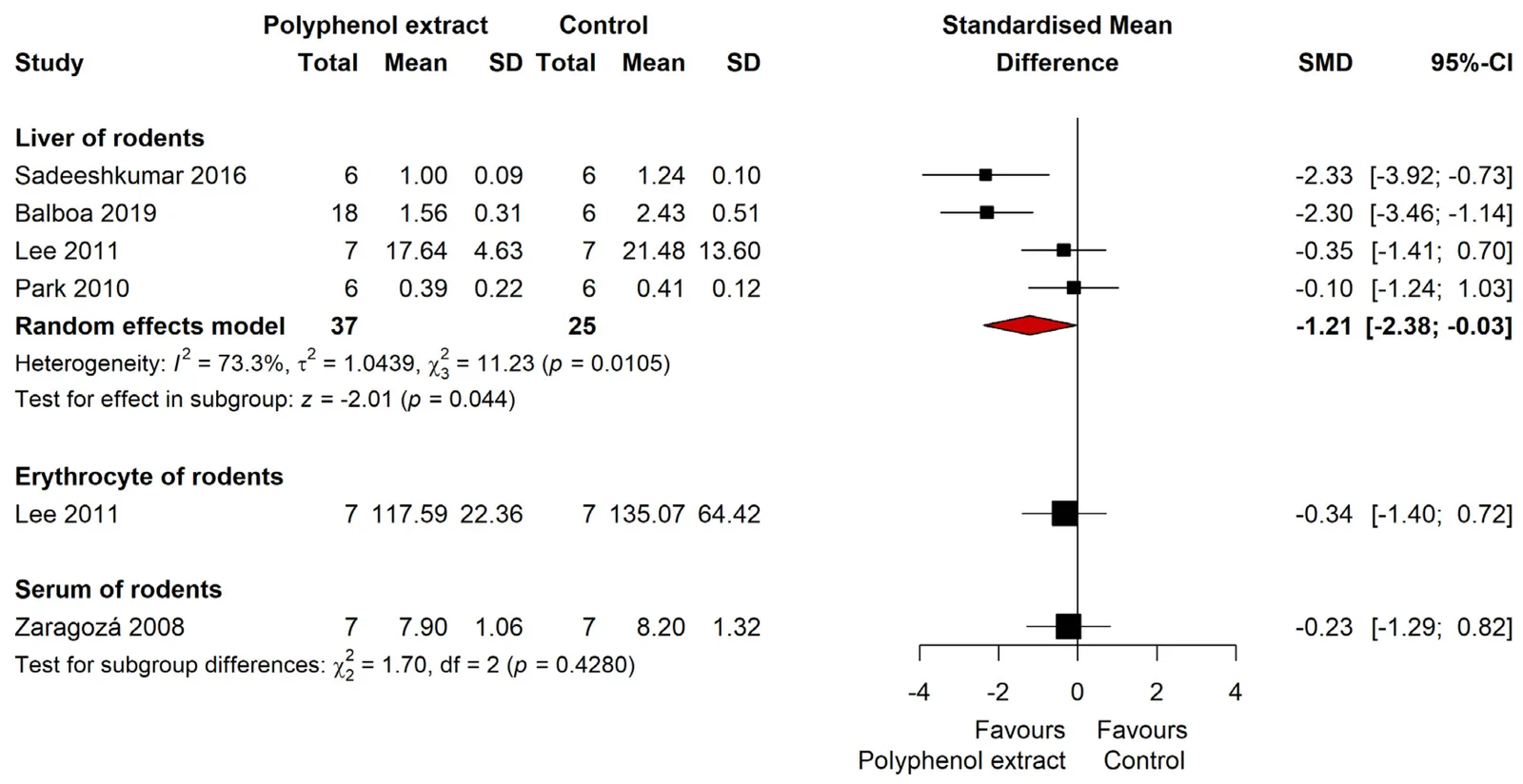
Forest plot of TBARS levels in rodent models by sample type [26,30,32,35,48].

For TBARS in rodents, the pooled effect in liver significantly favours polyphenol extract (SMD −1.21, 95% CI −2.38 to −0.03; *p* = 0.044), although heterogeneity is substantial (I^2^ = 73%). In contrast, erythrocyte (single study SMD −0.34, 95% CI −1.40 to 0.72) and serum (single study SMD −0.23, 95% CI −1.29 to 0.82) do not show clear differences. The test for subgroup differences is not significant (*p* = 0.428) (Figure 11).

**Figure 12:**
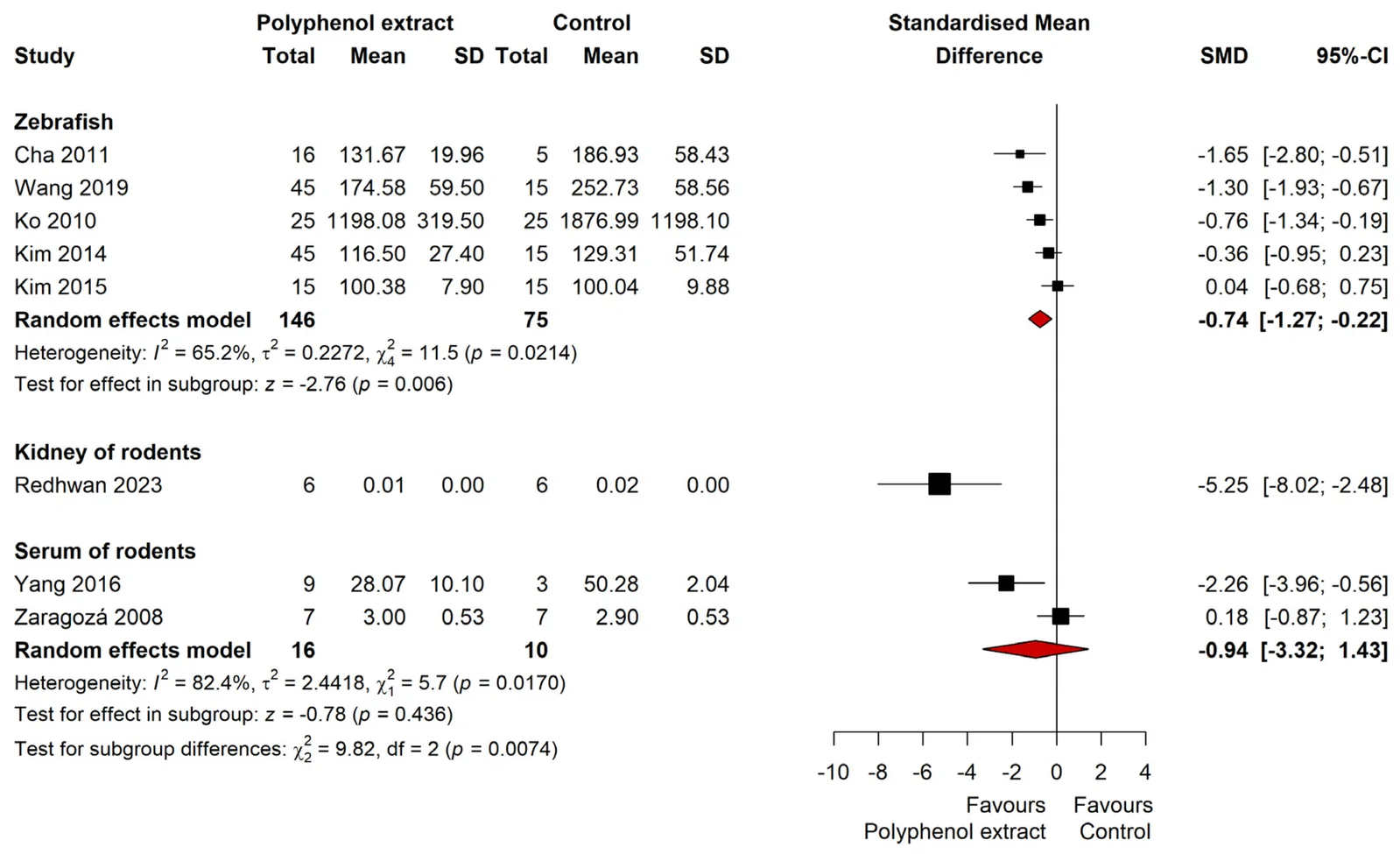
Forest plot of NO production subgrouped by model/organism [6,27,29,31,36,37,38,47].

For NO production, subgrouping by model shows a clear reduction in zebrafish (SMD −0.74, 95% CI −1.27 to −0.22; *p* = 0.006) with moderate heterogeneity (I^2^ = 65%). In rodents, the evidence is sparse and inconsistent as kidney shows a very large decrease (single study SMD −5.25, 95% CI −8.02 to −2.48), while serum pooled across two small studies is non-significant (SMD −0.94, 95% CI −3.32 to 1.43) with high heterogeneity (I^2^ = 82%). Overall, subgroup differences are significant (*p* = 0.0074) (Figure 12).

## 4. Discussion

This synthesis of in vivo evidence indicates that brown macroalgal polyphenols, particularly phlorotannins from *Ecklonia cava* and related taxa, consistently shift redox and inflammatory biology toward lower oxidative injury, with the clearest signal observed in zebrafish embryo models of acute oxidative stress. These findings suggest a robust and biologically coherent antioxidant effect under diverse experimental conditions.

To avoid ambiguity, this Discussion consistently distinguishes between: (1) direct evidence from the 22 studies included in our quantitative synthesis (phrased as ‘our meta-analysis showed’, ‘our results indicated’, or ‘the present synthesis’), and (2) inferential claims drawn from the broader or mechanistic literature on phlorotannins and oxidative stress (phrased as ‘the broader literature’, ‘contemporary phlorotannin literature’, ‘mechanistic literature’, or attributed to specific external studies).

Our zebrafish meta-analysis showed a large standardized reduction in ROS with very high between-study heterogeneity; however, the direction of effect persisted across leave-one-out analyses and across most compound-based subgroups. Importantly, the stability of the effect across sensitivity analyses strengthens the confidence in the validity of the observed association, despite heterogeneity. This pattern aligns with the broader marine polyphenol literature, which describes phlorotannins as multi-target modulators that couple direct radical quenching with inducible cytoprotective signaling [49,50].

The magnitude and consistency of ROS suppression in zebrafish are biologically coherent for the following three reasons. First, waterborne exposure yields rapid uptake and high effective exposure at early developmental stages, thereby enhancing the sensitivity of redox-responsive fluorescent assays [18,19]. Second, many zebrafish paradigms represent intense, short-latency stressors, such as UVB, AAPH, ethanol, hydrogen peroxide, and hyperglycemia, which induce acute oxidative bursts that are particularly modulated by phenolic compounds [51,52]. Third, several phlorotannins have sufficient electrophilic and redox-active chemistry to alter redox signaling nodes rather than only acting as stoichiometric antioxidants, which may explain the lower heterogeneity observed for purified compounds compared to complex extracts [53,54].

Mechanistically, the most frequently invoked axes in contemporary phlorotannin literature are Nrf2 driven antioxidant response programs and suppression of NF kB-mediated inflammation [7,14,55]. Reviews and recent experimental work converge on increased expression of phase II and antioxidant enzymes downstream of Nrf2, including heme oxygenase 1 and NADPH quinone oxidoreductase 1, alongside reduced inducible nitric oxide synthase, cyclooxygenase 2, and inflammatory cytokines under the control of NF [7,15,56].

The interpretation of in vivo efficacy is further complicated by the limited information on pharmacokinetics. Only a minority of studies have assessed systemic exposure or metabolite profiles, representing a significant gap in the evidence base. Available data suggest that high-molecular-weight phlorotannins exhibit limited absorption and undergo extensive biotransformation, indicating that their biological effects may be mediated by metabolites rather than intact compounds.

Our stressor stratification is informative, as hyperglycemia models show a stable protective signal with minimal heterogeneity, whereas UVB shows large but imprecise effects and extreme heterogeneity. This is consistent with the fact that UVB injury engages multiple parallel mechanisms beyond ROS generation, including DNA damage responses, mitochondrial depolarization, calcium flux, and apoptosis, all of which may be variably influenced by the compound identity, dose, and timing [57,58]. A recent nutraceutical-focused review highlighted the skin photoprotection and anti-apoptotic effects of seaweed phenolics and phlorotannins, supporting the idea that the UVB subgroup could be particularly sensitive to formulation and exposure details [8,17].

Although the activation of Nrf2-dependent antioxidant signaling and suppression of NF-κB-mediated inflammation are frequently proposed as central mechanisms of phlorotannin action, direct evidence for these pathways has been inconsistently reported in the included studies. Many studies have relied primarily on oxidative stress and lipid peroxidation markers without parallel assessment of upstream transcriptional regulators or downstream target genes. Therefore, the mechanistic interpretations in the present synthesis should be regarded as partly inferential and based on integration with the broader literature rather than on uniform experimental verification within the analyzed dataset.

In rodents, the pattern is more heterogeneous and tissue-specific, as lipid peroxidation endpoint trends are more consistent toward lower injury in the liver than antioxidant enzyme activity endpoints across tissues. This divergence is consistent with the fact that lipid peroxidation reflects cumulative oxidative damage, whereas enzyme activity represents dynamic and context-dependent adaptive responses [59,60].

Several explanations may account for this variation. Ceiling effects in antioxidant enzyme systems may limit detectable increases under high oxidative stress conditions, whereas damage endpoints such as MDA and TBARS remain modifiable [61]. A recent 2024 phlorotannin-focused study on liver injury highlights Nrf2-linked regulation of redox homeostasis and illustrates how antioxidant enzyme responses can diverge from damage markers depending on the intensity of the insult and the timing of sample collection [62]. This could explain why hepatic endpoints show more consistent responses than peripheral tissues.

Another factor is organ-specific pharmacokinetics and exposure. Oral administration may lead to higher local concentrations in gut-associated compartments and the liver through first-pass metabolism, whereas the kidney, muscle, and other peripheral tissues are more influenced by circulating metabolites rather than the parent phlorotannins [20]. This is particularly relevant because higher-molecular-weight phlorotannins exhibit limited intestinal absorption and undergo extensive biotransformation and microbial degradation, shaping their systemic availability [11]. A 2024 rat pharmacokinetic study directly quantified key *Ecklonia cava* phlorotannins after intravenous and oral administration, demonstrating that systemic exposure is measurable but varies substantially across different phlorotannin structures [21].

Finally, the model and assay heterogeneity contributed to this variability. Rodent studies encompass diverse stressors and disease models, with different tissues sampled, assay platforms employed, and normalization strategies used. Such variations can amplify the heterogeneity in enzyme activity measurements, particularly when studies combine preventive and therapeutic interventions or samples at different time points relative to the oxidative challenge.

Kidney findings illustrate this complexity in detail. Our SOD results included a kidney signal in the opposite direction from other tissues, whereas other kidney endpoints, such as NO and MDA, were sparse and highly variable. A mechanistic explanation is that renal oxidative stress responses may involve maladaptive enzyme induction, altered mitochondrial redox cycling, or differential inflammatory infiltration, depending on the nephrotoxin and the stage of injury [63]. In this context, recent studies have begun to target the upstream drivers of diabetic nephrotoxicity, including methylglyoxal and AGEs RAGE signaling [64]. A recent study on phlorofucofuroeckol A reported attenuation of methylglyoxal-induced nephrotoxicity via modulation of RAGE and advanced glycation pathways, providing a disease-relevant mechanism that could explain why simple enzyme activity changes do not always correlate with injury protection [65].

Our meta-analysis of NO suggests a clearer reduction in zebrafish than in rodents, likely reflecting the sharper inflammatory phenotype and standardized exposure in zebrafish assays [18]. In mammalian tissues, NO can be driven by multiple cell types and can fluctuate with perfusion and immune recruitment; therefore, sparse evidence may appear inconsistent [66]. However, mechanistic studies support phlorotannin suppression of inducible nitric oxide synthase and other inflammatory mediators through the NF and MAPK pathways. For example, diphlorethohydroxycarmalol has been shown to act on inflammatory signaling in muscle-related inflammation, consistent with enzyme and oxidative injury signals having partial tissue specificity [67].

A key conceptual point is the bidirectional crosstalk between Nrf2 and NF-kappaB programs [68]. The bidirectional crosstalk between antioxidant and inflammatory signaling likely explains the coordinated reduction in oxidative and inflammatory markers observed in previous studies. The induction of heme oxygenase 1 and other Nrf2 targets can dampen inflammatory signaling, whereas suppression of inflammatory transcription can reduce secondary ROS generation from immune enzymes [69]. Contemporary immunology reviews of the Nrf2 heme oxygenase 1 system support this integrated view and help interpret why endpoints such as ROS, lipid peroxidation, cytokines, and apoptosis often move toward tissue protection [56].

In reviews of marine algae bioactives, phlorotannins are repeatedly positioned as candidates for nutraceutical and skin-related applications because of their combined antioxidant, anti-inflammatory, and barrier-protective effects [8]. Topical delivery is particularly attractive when systemic bioavailability is limited. A previous mouse study demonstrated the mitigation of radiation dermatitis with topical phlorotannins, directly supporting a route-specific strategy that bypasses oral absorption constraints [25]. A recent cosmeceutical-focused review similarly cataloged formulation opportunities and dermatologic endpoints relevant to oxidative and inflammatory skin injury [8].

From a translational perspective, phlorotannins show promise as nutraceutical and cosmeceutical agents. However, the predominance of preventive study designs limits direct clinical applicability, particularly in established disease contexts. Nevertheless, recent advances in formulation and delivery strategies may help bridge this gap. At the same time, the translational narrative increasingly includes delivery innovation. A 2024 study on *Ecklonia cava*-derived extracellular vesicles in aged mice proposes downregulation of MAPK and NF-κB signaling together with improvements in skin aging endpoints, suggesting that vesicle-based delivery or co-delivered bioactives may expand the therapeutic window for cutaneous indications [12].

### 4.1. Limitations

Several limitations constrain the inferences and help explain the heterogeneity. First, reporting quality and internal validity safeguards were frequently unclear in SYRCLE domains aligned with randomization, allocation concealment, and blinding, which increases the risk of bias and inflates effect sizes in preclinical intervention literature. This issue is not unique to this field and has motivated updated reporting standards, including ARRIVE 2.0, which would materially improve reproducibility and enable higher confidence meta-analytic synthesis [22]. Previous meta-research in preclinical studies has demonstrated that inadequate reporting and a lack of internal validity safeguards are associated with exaggerated treatment effects. Newer appraisal frameworks for preclinical studies also highlight how incomplete reporting limits interpretability, even when downstream outcome handling appears adequate [70]. Second, exposure characterization was heterogeneous, and dosing regimens were inconsistent across the included studies. The extracts vary in phlorotannin composition, degree of polymerization, and co-extracted constituents, and only a minority of reports provide a detailed chemical characterization of polyphenol-rich extracts or fractions. While several studies have quantified total phenolic or phlorotannin content using colorimetric assays, comprehensive profiling by HPLC, LC–MS, or NMR has been inconsistently reported, and the relative abundance of individual phlorotannins and their degree of polymerization could not be systematically compared. This limited chemical standardization restricts the interpretation of dose–response relationships and complicates the attribution of biological effects to specific compounds. Moreover, the administered doses ranged from micromolar concentrations in zebrafish immersion models to gram-per-kilogram oral doses in rodents, spanning several orders of magnitude. Such wide dose ranges hinder direct comparisons between experimental systems and raise questions regarding physiological relevance and translational feasibility. In several rodent studies, the doses used substantially exceeded the levels that would be realistically achievable through dietary intake or supplementation in humans. Therefore, a systematic evaluation of dose–response relationships and the establishment of clinically relevant exposure ranges remain important unmet needs. Third, some primary outcomes rely on assays with known, interpretive constraints. Fluorescent probes used for ROS, including DCF-based approaches, can be influenced by multiple oxidant species and experimental conditions, which can amplify the heterogeneity across zebrafish studies, even when the direction of effect is stable [71]. Fourth, most designs are preventive rather than therapeutic, limiting their direct relevance to clinical scenarios where treatment follows an established disease or injury.

Fifth, the included evidence base is characterized by a marked geographic concentration, with the majority of studies originating from South Korea, supplemented by contributions from China, India, and Spain. This regional clustering may introduce site-specific research practices, algae sourcing patterns, and standardization approaches that limit generalizability to other geographic and ecological contexts where brown macroalgae species composition, cultivation conditions, and phytochemical profiles may differ substantially. Sixth, sample sizes across primary studies are frequently small, with many experimental groups containing as few as three to six animals, particularly in rodent studies employing disease models such as NDEA hepatocarcinogenesis, radiation injury, or glucocorticoid-induced osteoporosis. These limited sample sizes reduce statistical power, increase susceptibility to random error, and may inflate effect estimates, especially when combined with the high between-study heterogeneity observed across forest plots for outcomes, including ROS generation, catalase activity, and SOD production.

Seventh, the taxonomic diversity of the experimental models introduces translational uncertainty. While zebrafish embryos provide valuable high-throughput screening data with standardized fluorescent readouts, the evolutionary distance between teleost fish and mammals encompasses substantial differences in antioxidant defense systems, xenobiotic metabolism, and tissue-specific pharmacokinetics. The marked heterogeneity observed in subgroup analyses comparing zebrafish and rodent endpoints suggests that the effects of phlorotannins may operate through distinct mechanisms or with different magnitudes across vertebrate classes, complicating direct extrapolation to mammalian physiology. Eighth, the tissue-specific findings were heterogeneous and occasionally contradictory. For instance, SOD activity shows increases in the serum and liver but significant decreases in the kidney, while MDA reductions are more consistent in hepatic tissue than in renal or systemic compartments. This tissue-dependent variability may reflect organ-specific uptake, differential enzymatic transformation, or context-dependent redox signaling; however, systematic investigations to establish predictive patterns for target tissue engagement are lacking.

Ninth, temporal dynamics and sampling timing are inconsistently reported or vary across studies, preventing the determination of optimal intervention windows and limiting the understanding of whether observed effects represent acute scavenging versus sustained pathway modulation. Tenth, although leave-one-out sensitivity analyses confirmed that the pooled effects remained directionally consistent, high I^2^ values exceeding 80% persisted across multiple outcomes, including ROS generation, catalase activity, and SOD production, indicating substantial unexplained heterogeneity that random-effects models cannot fully accommodate. Finally, the relatively small number of primary studies for several outcomes limits the reliability of funnel plot asymmetry assessments and Egger’s regression tests, constraining confidence in the absence of publication bias as a complete explanation for observed effect sizes and emphasizing the need for larger-scale primary research to clarify the true magnitude and consistency of phlorotannin effects across experimental conditions.

### 4.2. Future Directions

Progress toward translational readiness can benefit from several concrete priorities. First, harmonizing models and endpoints is essential, as researchers should pre-specify core oxidative injury endpoints alongside adaptive enzyme endpoints, including time-course sampling, and standardize tissue processing and normalization. Improving chemical and exposure standardization is also critical, with quantitative profiling of major phlorotannins in extracts and confirmation of stability in dosing vehicles, particularly for waterborne zebrafish and dietary rodent exposures. Mechanistic validation should be pursued using pathway perturbation approaches, including Nrf2 loss-of-function models or pharmacological blockades, and by measuring downstream targets, such as heme oxygenase 1, NQO1, and inflammatory transcriptional markers, to link observed phenotypes to causal signaling. Clarifying ADME and metabolite contributions is important as well; pharmacokinetic mapping should extend beyond parent compounds and integrate microbiome-derived metabolites, given limited oral bioavailability and potential generation of bioactive small molecules through microbial transformation. Shifting toward therapeutic designs will help determine whether phlorotannins can modify established injury rather than solely prevent its development by incorporating delayed dosing paradigms and clinically relevant comparators. Finally, early-phase human studies aligned with biomarkers should be prioritized, focusing on indications with measurable oxidative stress markers, such as metabolic dysregulation or inflammatory skin injury, while carefully monitoring iodine and contaminant-related safety considerations highlighted in functional food reviews.

### 4.3. Implications

Overall, our results support a model in which phlorotannins act as network modulators of redox and inflammation, with the strongest and most reproducible evidence in zebrafish acute stress paradigms and in rodent liver lipid peroxidation endpoints. The zebrafish platform appears to be well-suited for screening compound classes and stressor specificity, whereas rodent studies are necessary to resolve tissue distribution, disease relevance, and dosing feasibility. Contemporary reviews in marine polyphenols and seaweed phenolics contextualize these findings within a broader landscape of cardiometabolic and anti-inflammatory potential but also note that clinical translation remains limited by standardization and human evidence gaps [16].

## 5. Conclusions

Preclinical evidence indicates that brown algal phlorotannins consistently reduce oxidative stress-related injury signals, especially reactive oxygen species (ROS) and lipid peroxidation, and frequently downshift inflammatory mediators through convergent Nrf2 centered cytoprotection and NF-kappaB-centered inflammatory suppression. Confidence in the effect size and tissue specificity is limited by heterogeneity in models, formulations, endpoints, and reporting quality. Better chemical standardization, ARRIVE aligned study design, and mechanistic and pharmacokinetic integration, followed by biomarker anchored human studies, are the most direct steps to advance nutraceutical and cosmeceutical applications.

## Figures and Tables

**Table 1:** Summary characteristics of the included studies.

Study ID	Animal				Intervention					Control Group	Conclusion
	Species	Age, Weight	Sex	Number per Study Group	Brown Macroalgae Species	Polyphenol Type	Dose, Frequency	Timing Related to the Stressor (Preventive or Therapeutic)	Duration of Exposure		
Balboa 2019 [48]	Sprague-Dawley rats	NR, 90–100 g	Male	6	*Sargassum muticum*	Phlorotannin crude extract	0.5, 1, or 2 g/kg, Daily	-	3 weeks	Standard diet	*Sargassum muticum* can be processed by autohydrolysis and membrane ultrafiltration to obtain fucoidan- and phlorotannin-rich extracts with low mineral content, Trolox-equivalent antiradical activity, and in vivo antioxidant effects, evidenced by reduced serum glucose, hepatic GPx, and TBARS levels without changes in body or organ weights.
Cha 2012 [47]	Zebrafish (*Danio rerio*)	2 dpf embryos	NA	25 totally	*Ecklonia cava*	Phloroglucinol, Eckol, Triphlorethol, or Eckstolonol	50 μM	Preventive	6 h	No UVB or UVB-only	Phlorotannins isolated from *Ecklonia cava* exhibited significant photoprotective effects against UV-B exposure in a zebrafish embryo model, with good tolerability and no observed adverse effects. While zebrafish embryos are a validated vertebrate in vivo model with skin architecture comparable to mammals, further studies are required to confirm safety and efficacy in humans and domestic animals.
Cha 2017 [46]	Zebrafish (*Danio rerio*)	7–9 hpf (start) or 3 dpf (MNZ treatment)	NA	10–13	*Ecklonia cava*	Phloroglucinol	50 µM (for H_2_O_2_) or 400 µM (for MNZ)	Preventive for H_2_O_2_ and therapeutic for MNZ	120 hpf for H_2_O_2_ 24 h for MNZ	No stress, H_2_O_2_ only, or MNZ only	The findings indicate that phloroglucinol isolated from *Ecklonia cava* effectively protects against H_2_O_2_-induced hepatic injury and promotes liver regeneration, supporting its potential use as a nutra-pharmaceutical ingredient for liver-related disorders.
Chen 2022 [45]	Zebrafish (*Danio rerio*)	2 dpf embryos	NA	30	*Sargassum thunbergii*	Polyphenol-rich fraction	0.8, 1.2, or 1.6 μg/mL	Preventive	24 h	No UVB or UVB-only	STPE attenuated UVB-induced oxidative stress, inflammatory cytokine expression, and DNA condensation through suppression of the NF-κB signaling pathway, indicating a protective effect against UVB-mediated photodamage and potential for clinical development in skin disease therapy.
Kang 2012a [44]	BALB/c mice	6 weeks, 20–25 g	Male	9	*Ecklonia cava*	Dieckol	5, or 25 mg/kg	Preventive	2 weeks	Saline or Ethanol only	This study demonstrated that DRP protects against ethanol-induced liver injury in vivo, suggesting potential hepatoprotective benefits against alcohol-related liver damage.
Kang 2012b [43]	Zebrafish (*Danio rerio*)	3 dpf embryos	NA	25	*Ecklonia cava*	Dieckol, Eckstolonol, Eckol, Triphlorethol, or Phloroglucinol	50 uM	Preventive	120 h	No AAPH or AAPH only	Phlorotannins isolated from *Ecklonia cava* effectively inhibited AAPH-induced intracellular ROS generation, lipid peroxidation, and cell death. In a zebrafish embryo model, these compounds attenuated AAPH-induced toxicity, supporting the utility of zebrafish as an in vivo model for evaluating the antioxidant effects of natural compounds.
Kang 2012c [42]	C57BL/KsJ-db/db mice	9 weeks, 50–55 g	Male	7	*Ecklonia cava*	Dieckol	10, or 20 mg/kg	Therapeutic	2 weeks	Saline treatment	The findings indicate that dieckol, isolated from the brown seaweed *Ecklonia cava*, shows potential for development as a natural nutraceutical for the prevention of type II *diabetes mellitus*.
Kang 2013a [41]	ICR mice	6 weeks, 25–30 g	Male	5	*Ecklonia cava*	Dieckol	5, or 25 mg/kg	Preventive	One week	Saline or CCl_4_ only	Dieckol isolated from *Ecklonia cava* exerts hepatoprotective effects by modulating apoptosis-related pathways, characterized by upregulation of Bcl-xL and downregulation of Bax in hepatic tissue, suggesting potential utility in preventing CCl_4_-induced liver injury.
Kang 2013b [40]	Zebrafish (*Danio rerio*)	7–9 hpf	NA	25	*Ecklonia cava*	Dieckol	10, or 20 μM	Preventive	Exposure up to 24 hpf	No ethanol or Ethanol only	Dieckol isolated from *Ecklonia cava* may exert protective effects against ethanol-induced liver disease.
Kang 2014 [39]	Zebrafish (*Danio rerio*)	3 dpf embryos	NA	25	*Ishige foliacea*	Octaphlorethol A	25, 50, or 100 μM	Preventive	Exposure up to 120 hpf	No glucose or Glucose only	The findings indicate that OPA mitigates high-glucose–induced oxidative damage by suppressing ROS production, lipid peroxidation, and cell death in vitro in HUVECs and in vivo in a zebrafish model.
Kim 2014 [38]	Zebrafish (*Danio rerio*)	7–9 hpf	NA	15	*Ecklonia cava* processing by-product	Polyphenol-rich fraction (PRF)	25, 50, or 100 μg/mL	Preventive	Exposure up to 24 hpf	No LPS or LPS only	The results show that PRF inhibited NO production and reduced iNOS and COX-2 expression in LPS-stimulated RAW264.7 cells, and attenuated ROS and NO generation as well as cell death in an LPS-induced zebrafish inflammation model. These findings suggest that EPB by-products from seaweed processing may represent a potential source of anti-inflammatory agents for inflammatory disease treatment.
Kim 2015 [37]	Zebrafish (*Danio rerio*)	7–9 hpf	NA	15	*Ecklonia cava*	6,6-bieckol, Phloroeckol, Dieckol, or Phlorofucofuroeckol-A	20 μM	Preventive	Exposure up to 48 hpf	No glucose or Glucose only	The study demonstrated that DK confers protection against high-glucose–induced oxidative stress in a zebrafish model, supporting the utility of this model for elucidating underlying mechanisms and for identifying functional antioxidants relevant to food and nutraceutical applications.
Ko 2010 [36]	Zebrafish (*Danio rerio*)	2 hpf	NA	25	*Ecklonia cava*	Dieckol	50 μM	Preventive	-	No UVB or UVB-only	The results indicate that dieckol possesses a higher number of functional hydroxyl groups compared with other phlorotannins, supporting its potential application as a natural marine-derived compound in antioxidant and cosmeceutical industries.
Lee 2012 [35]	C57BL/KsJ-db/db mice	5 weeks	Male	7	*Ecklonia cava*	Dieckol	0.5 g/100 g diet	Therapeutic	6 weeks	Standard semisynthetic diet only	These findings suggest that AG-dieckol exerts antidiabetic effects in a type 2 diabetic mouse model by improving glucose and lipid metabolism and enhancing antioxidant enzyme activity.
Lee 2015 [34]	Zebrafish (*Danio rerio*)	12 hpf	NA	NR	*Ecklonia cava*	Phlorofucofuroeckol-A	5, 20.8, or 41.5 μM	Preventive	-	No AAPH or AAPH only	The study demonstrated that PFF-A isolated from *Ecklonia cava* exhibits strong protective effects against AAPH-induced lipid peroxidation both in vitro and in vivo, suggesting its potential use as a functional supplement targeting lipid peroxidation.
Lee 2017 [33]	Zebrafish (*Danio rerio*)	NR	NA	NR	*Ishige foliacea*	Octaphlorethol A	3, 6, 12.5, or 25 µg/mL	Preventive	-	No AAPH or AAPH only	An efficient preparative CPC–ABTS^+^ on-line HPLC approach was established for high-yield isolation of OPA from the EtOAc fraction of *Ishige foliacea*, and the isolated compound exhibited pronounced antioxidant activity in an AAPH-induced zebrafish embryo model.
Park 2010 [32]	C57BL/6 mice	6–8 weeks, 18–25 g	NR	3–6	*Ecklonia cava*	Dieckol	10, or 25 mg/kg bw	Preventive	16 h	A sham irradiated group, or an irradiated control group	Dieckol can be a promising agent for reducing the time needed for reconstitution of hemopoietic cells after exposure to irradiation.
Redhwan 2023 [31]	Wistar rats	NA, 150–200 gm	NR	6	*Ecklonia cava*	Polyphenol-rich fraction	200 mg/kg b.w	Preventive	4 weeks	No KBrO_3_, or KBrO_3_ only	The findings suggest that ECPP acts as an in vivo antioxidant by scavenging reactive oxygen species, thereby mitigating KBrO_3_-induced oxidative renal injury in rats.
Sadeeshkumar 2016 [30]	Wistar rats	NA, 150–170 gm	Male	6	*Ecklonia cava*	Dieckol	10, 20, or 40 mg/kg bw	Preventive	15 weeks	Saline or NDEA only	DK treatment significantly reduced tumor incidence in NDEA-initiated hepatocarcinogenesis in rats and attenuated hepatic oxidative damage by lowering liver marker enzymes and lipid peroxidation while enhancing antioxidant defenses. These findings suggest that DK has potential for development as a chemotherapeutic agent, warranting further investigation of its underlying molecular mechanisms and anticancer efficacy.
Wang 2019 [29]	Zebrafish (*Danio rerio*)	2 hpf	NA	15	*Ishige okamurae*	Diphlorethohydroxycarmalol (DPHC)	25, 50, or 100 μM	Preventive	6 h	No UVB or UVB-only	DPHC exhibits strong photoprotective effects in vitro and in vivo, supporting its potential application as an ingredient in pharmaceutical and cosmeceutical products.
Wang [28] 2022	Sprague-Dawley rats	20–24 weeks, 22–25 g	Female	10	*Ecklonia cava*	Dieckol	50 mg/kg	Therapeutic	20 weeks	No glucocorticoid or glucocorticoid only	Dieckol modulated body and reproductive organ weights, improved bone microarchitecture, hormonal and nutritional status, enhanced endogenous antioxidant levels, and reduced inflammation, supporting its potential as a prophylactic agent against glucocorticoid-induced osteoporosis.
Yang 2016 [6]	C57BL/6 mice	6 weeks, NA	Male	8	*Ecklonia cava*	Dieckol	10, 50, or 100 mg/kg/day	Preventive	1 week	No LPS or LPS only	The study indicates that *Ecklonia cava* extract and its major phlorotannin, dieckol, may attenuate septic shock by negatively regulating pro-inflammatory mediators through the NF-κB and Nrf2/HO-1 signaling pathways, supporting the potential nutraceutical relevance and health benefits of brown algal phlorotannins.
Yang 2020 [25]	BALB/c mice	5 weeks, NA	Female	≥ 5	*Ecklonia cava*	Phlorotannins (PTNs) solution	0.05%, or 0.5%	Preventive	3 weeks	Sham, or Radiation only	The study demonstrated that topical PTN application ameliorated radiation dermatitis in a mouse model by activating anti-inflammatory and antioxidant signaling pathways. Given the lack of effective treatments for radiation dermatitis in patients undergoing radiotherapy, these findings suggest the potential use of PTNs as cosmeceutical agents to prevent or reduce radiation-induced skin damage.
Zaragozá 2008 [27]	Swiss mice and Sprague-Dawley rats	NA, 150 g	Male and female	14	*Fucus vesiculosus*	Polyphenol-rich fraction	200 or 750 mg/kg	-	4 weeks	1% carboximethylcelulose	The study demonstrates that two extracts from *Fucus vesiculosus* are non-toxic and exhibit antioxidant activity against chemically generated oxidants in vitro, while only extract 2 shows ex vivo antioxidant efficacy by inhibiting oxidant formation, scavenging superoxide anions, and reducing reactive intermediates. The findings support the potential application of extract 2 (Healsea) as a functional food or dietary supplement, with possible relevance to human antioxidant activity despite limitations in extrapolating animal data to humans.
Zhao 2016 [26]	Kunming mice	4 weeks, 8–22 g	Male	10	*Sargassum hemiphyllum*	Crude phlorotannins extract	7.5, 15, or 30 mg/kg	Preventive	4 weeks	Distilled water, or Distilled water plus the CCl_4_	The results provide evidence that CPEs extracted from SH exhibit antioxidant activity in vitro and in vivo, effectively scavenging free radicals and protecting the liver, kidney, and brain from CCl_4_-induced oxidative damage.

AAPH = 2,2′-azobis(2-methylpropionamidine) dihydrochloride (a water-soluble azo radical initiator used to induce oxidative stress); ABTS = 2,2′-azino-bis(3-ethylbenzothiazoline-6-sulfonic acid) (a stable radical cation employed in antioxidant capacity assays); AG-dieckol = a specific grade/formulation of dieckol as used in Lee 2012 [35]; AIN-93G = American Institute of Nutrition-93 Growth diet (a purified standard rodent diet); bw/b.w. = body weight; CCl_4_ = carbon tetrachloride; CMC = carboxymethyl cellulose; COX-2 = cyclooxygenase-2; CPC = centrifugal partition chromatography; CPEs = crude phlorotannin extracts; DK = dieckol; DMSO = dimethyl sulfoxide; dpf = days post-fertilization (zebrafish embryos); DPHC = diphlorethohydroxycarmalol (a phlorotannin from *Ishige okamurae*); DRP = dieckol-rich phlorotannins; ECPP = *Ecklonia cava* polyphenol preparation; EPB = *Ecklonia cava* processing by-product; EtOAc = ethyl acetate; GPx = glutathione peroxidase; H_2_O_2_ = hydrogen peroxide; hpf = hours post-fertilization (zebrafish embryos); HPLC = high-performance liquid chromatography; HUVECs = human umbilical vein endothelial cells; ICR = Institute of Cancer Research (outbred albino mouse strain); iNOS = inducible nitric oxide synthase; KBrO_3_ = potassium bromate; Kunming = an outbred albino mouse strain of Chinese origin; LPS = lipopolysaccharide; mJ/cm^2^ = millijoules per square centimeter (unit of UV-B irradiance); MNZ = metronidazole; NA = not available/not applicable; NDEA = N-nitrosodiethylamine; NF-κB = nuclear factor kappa-light-chain-enhancer of activated B cells; NO = nitric oxide; NR = not reported; OPA = octaphlorethol A (a phlorotannin from *Ishige* sp.); PBS = phosphate-buffered saline; PFF-A = phlorofucofuroeckol-A (a phlorotannin from *Ecklonia cava*); PRF = polyphenol-rich fraction; PTNs = phlorotannins; ROS = reactive oxygen species; SH = *Sargassum hemiphyllum* (acronym used in Zhao 2016 [26]); STPE = *Sargassum thunbergii* polyphenol extract; TBARS = thiobarbituric acid reactive substances; Trolox = 6-hydroxy-2,5,7,8-tetramethylchroman-2-carboxylic acid; UV-B = ultraviolet B radiation (280–315 nm); µg/mL = micrograms per milliliter; μM = micromolar (micromoles per liter).

## Data Availability

The data supporting the conclusions of this article are included within the article and its additional files.

## Supplementary Materials

The following supporting information can be downloaded at https://www.mdpi.com/article/10.3390/antiox15080961/s1, Table S1: Search strategy; Table S2: Detailed characteristics of the included studies; Figure S1: Funnel plot for the zebrafish ROS meta-analysis with Egger’s test for small-study effects; Figure S2: Subgroup meta-analysis of zebrafish ROS by brown macroalgae species; Figure S3: Subgroup meta-analysis of zebrafish ROS by stressor/model; Figure S4: Species-stratified (rats vs. mice) subgroup analysis of rodent CAT activity by sample type; Figure S5: Species-stratified (rats vs. mice) subgroup analysis of rodent GPx activity by sample type; Figure S6: Species-stratified (rats vs. mice) subgroup analysis of rodent MDA levels by sample type; Figure S7: Species-stratified (rats vs. mice) subgroup analysis of rodent SOD activity by sample type.

## Abbreviations

The following abbreviations are used in this manuscript:

AAPH	2,2′-Azobis(2-amidinopropane) dihydrochloride
CAT	Catalase
DPHC	Diphlorethohydroxycarmalol
DPPP	Diphenyl-1-pyrenylphosphine
GPx	Glutathione peroxidase
LPS	Lipopolysaccharide
MDA	Malondialdehyde
MNZ	Metronidazole
NDEA	N-Nitrosodiethylamine
NO	Nitric oxide
PRF	Polyphenol-rich fraction
PTNs	Phlorotannins
ROS	Reactive oxygen species
SOD	Superoxide dismutase
TBARS	Thiobarbituric acid reactive substances
