# Supplementary material for: Brown Macroalgal Polyphenols and Oxidative Stress: A Systematic Review and Meta-Analysis of In Vivo Evidence in Vertebrate Models
Source: Antioxidants (Basel). 2026 Jul 31;15(8):961. doi: 10.3390/antiox15080961 (PMC13509805; doi:10.3390/antiox15080961)
Supplement: Supplementary file 1 [file antioxidants-15-00961-s001.zip › antioxidants-4449766-supplementary.pdf]

Supplementary Table S1: Search strategy.

Database	Search term	Filters	Results
PubMed	(polyphenols OR phlorotannins OR Phloroglucinol OR Eckol OR Triphlorethol OR Eckstolonol OR Dieckol OR 6,6-bieckol) AND (brown algae OR seaweed OR macroalgae) AND (oxidative stress) AND (animal model OR rat OR mouse OR mice OR zebrafish OR in vivo)	All field	63
Scopus	(polyphenols OR phlorotannins OR Phloroglucinol OR Eckol OR Triphlorethol OR Eckstolonol OR Dieckol OR 6,6-bieckol) AND (brown algae OR seaweed OR macroalgae) AND (oxidative stress) AND (animal model OR rat OR mouse OR mice OR zebrafish OR in vivo)	All field	203
WOS	(polyphenols OR phlorotannins OR Phloroglucinol OR Eckol OR Triphlorethol OR Eckstolonol OR Dieckol OR 6,6-bieckol) AND (brown algae OR seaweed OR macroalgae) AND (oxidative stress) AND (animal model OR rat OR mouse OR mice OR zebrafish OR in vivo)	All field	147
Cochrane	(polyphenols OR phlorotannins OR Phloroglucinol OR Eckol OR Triphlorethol OR Eckstolonol OR Dieckol OR 6,6-bieckol) AND (brown algae OR seaweed OR macroalgae) AND (oxidative stress) AND (animal model OR rat OR mouse OR mice OR zebrafish OR in vivo)	All field	2

Supplementary Table S2. Detailed characteristics of the included studies.

Study ID	***Country***	***The time when the phenol is extracted***	***Stressor***	***Intervention***	
				Route	***Vehicle***
**Balboa 2019**	Spain	NR	None	Intragastric tube	Distilled water
**Cha 2011**	Korea	October 2007 to March 2008	UV-B (50 mJ/cm2)	Waterborne exposure (immersion)	Medium containing 0.1% DMSO
**Cha 2017**	Republic of Korea	October 2013 to March 2014	Liver damaged by H_2O_2 (5 mM) or Metronidazol e (MNZ, 15 mM)	Waterborne exposure (immersion)	Embryo medium
**Chen 2022**	China	NR	UV-B (20 mJ/cm2)	Waterborne exposure (immersion)	-
**Kang 2012a**	Republic of Korea	March to May 2009	Ethanol (4 g/kg) induced liver damage	Oral	Saline
**Kang 2012b**	Korea	October 2007 to March 2008	AAPH (25 mM)-induced oxidative stress	Waterborne exposure (immersion)	0.1% DMSO
**Kang 2012c**	Republic of Korea	NR	Type II diabetes	Intraperitoneal	Saline
**Kang 2013a**	Republic of Korea	NR	Carbon tetrachloride (CCl4) (0.5mg/kg, mouse)-induced acute liver damage	Oral	Saline
**Kang 2013b**	Republic of Korea	February to May 2012	Ethanol-induced cell damage and apoptosis	Waterborne exposure (immersion)	Embryo medium

**Kang 2014**	Republic of Korea	NR	High glucose (150 µM)-induced oxidative damage	Waterborne exposure (immersion)	0.1% DMSO
**Kim 2014**	Korea	NR	Lipopolysaccharide (LPS) (10 µg /mL)-induced inflammation	Waterborne exposure (immersion)	Embryo medium
**Kim 2015**	Republic of Korea	March 2012 to June 2012	High glucose (150 µM)-induced oxidative damage	Waterborne exposure (immersion)	Embryo medium
**Ko 2010**	Republic of Korea	NR	UV-B (50 mJ/cm2)	Waterborne exposure (immersion)	0.1% DMSO
**Lee 2011**	Republic of Korea	NR	Type II diabetes	Oral	Standard semisynthetic diet (AIN-93G)
**Lee 2015**	Korea	June 2013	AAPH (15 mM)-induced oxidative stress	Waterborne exposure (immersion)	Embryo medium
**Lee 2017**	Republic of Korea	April 2014	AAPH (15 mM)-induced oxidative stress	Waterborne exposure (immersion)	-
**Park 2010**	South Korea	NR	Lethal/Sublethal Ionizing Radiation	Intraperitoneal	Phosphate buffer saline (PBS)
**Redhwan 2023**	India	NR	Potassium Bromate (KBrO3-) (135 mg/kg b.w)-Induced Nephrotoxicity	Oral	-
**Sadeesh kumar 2016**	India	NR	N-nitrosodiethylamine (NDEA) induced hepatocarcinogenesis	Oral	Isotonic saline
**Wang 2019**	Republic of Korea	NR	UV-B (50 mJ/cm2)	Waterborne exposure (immersion)	-

**Wang 2022**	China	NR	Dexamethasone (2.5 mg/kg/day for 5 days) induced osteoporosis	Oral	1% carboxymethyl cellulose (CMC)
**Yang 2016**	South Korea	NR	Lipopolysaccharide (25 mg/kg)-Induced Sepsis	Oral	Phosphate buffer saline (PBS)
**Yang 2020**	Korea	NR	Radiation Dermatitis (X-rays, 36 Gy)	Topical	Sesame oil
**Zaragozá 2008**	Spain	NR	None	Oral (intragastric cannula) and Intraperitoneal	1% carboxymethylcellulose
**Zhao 2016**	China	NR	Carbon tetrachloride (CCl_4) (319 mg/kg, mouse)-induced acute liver damage	Intragastric	Distilled water

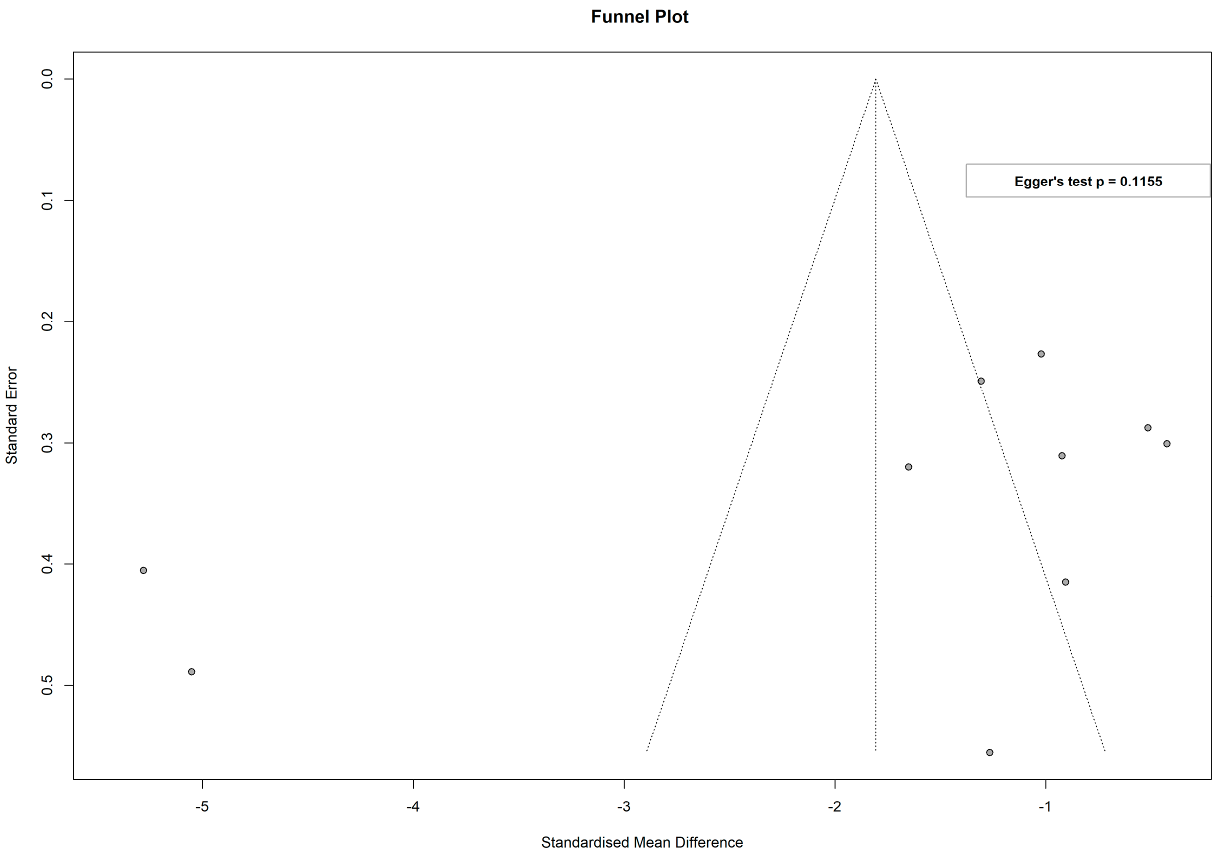

Supplementary Figure S1. *Funnel plot for the zebrafish ROS meta-analysis with Egger's test for small-study effects. Egger's regression test evaluates small-study effects by testing whether the intercept (β_o) of the weighted linear regression of the standardized effect size (yi / SEi) on precision (1 / SEi) differs significantly from zero. The test statistic follows a Student's t-distribution with k − 2 degrees of freedom:* ***$t = \beta_o / SE(\beta_o)$*** *where k is the number of studies, where $SE(\beta_0)$ is the standard error of the intercept estimated from the regression.*

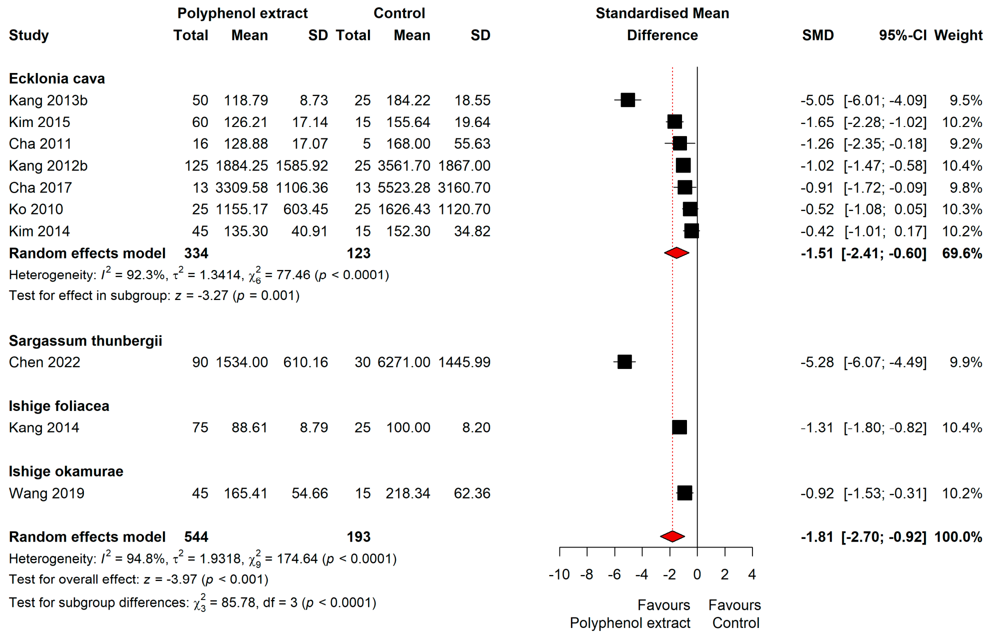

Supplementary Figure S2. *Subgroup meta-analysis of zebrafish ROS by brown macroalgae species*

Study	Polyphenol extract Total	Mean	SD	Control Total	Mean	SD	SMD	95%-CI	Weight
**UV-B**									
Chen 2022	90	1534.00	610.16	30	6271.00	1445.99	-5.28	[-6.07; -4.49]	9.9%
Cha 2011	16	128.88	17.07	5	168.00	55.63	-1.26	[-2.35; -0.18]	9.2%
Wang 2019	45	165.41	54.66	15	218.34	62.36	-0.92	[-1.53; -0.31]	10.2%
Ko 2010	25	1155.17	603.45	25	1626.43	1120.70	-0.52	[-1.08; 0.05]	10.3%
**Random effects model**	**176**			**75**			**-1.99**	**[-4.09; 0.11]**	**39.5%**
Heterogeneity: $I^2 = 97\%$, $\tau^2 = 4.4264$, $\chi^2_3 = 101.67$ ($p < 0.0001$)									
Test for effect in subgroup: $z = -1.86$ ($p = 0.063$)									
**Liver damaged by H2O2**									
Cha 2017	13	3309.58	1106.36	13	5523.28	3160.70	-0.91	[-1.72; -0.09]	9.8%
**AAPH**									
Kang 2012b	125	1884.25	1585.92	25	3561.70	1867.00	-1.02	[-1.47; -0.58]	10.4%
**Ethanol**									
Kang 2013b	50	118.79	8.73	25	184.22	18.55	-5.05	[-6.01; -4.09]	9.5%
**High glucose**									
Kim 2015	60	126.21	17.14	15	155.64	19.64	-1.65	[-2.28; -1.02]	10.2%
Kang 2014	75	88.61	8.79	25	100.00	8.20	-1.31	[-1.80; -0.82]	10.4%
**Random effects model**	**135**			**40**			**-1.44**	**[-1.82; -1.05]**	**20.5%**
Heterogeneity: $I^2 = 0\%$, $\tau^2 = 0$, $\chi^2_1 = 0.72$ ($p = 0.3969$)									
Test for effect in subgroup: $z = -7.30$ ($p < 0.001$)									
**LPS**									
Kim 2014	45	135.30	40.91	15	152.30	34.82	-0.42	[-1.01; 0.17]	10.2%
**Random effects model**	**544**			**193**			**-1.81**	**[-2.70; -0.92]**	**100.0%**
Heterogeneity: $I^2 = 94.8\%$, $\tau^2 = 1.9318$, $\chi^2_9 = 174.64$ ($p < 0.0001$)									
Test for overall effect: $z = -3.97$ ($p < 0.001$)									
Test for subgroup differences: $\chi^2_5 = 70.57$, df = 5 ($p < 0.0001$)									

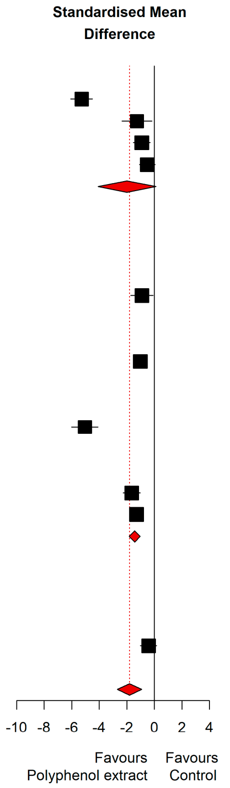

Supplementary Figure S3. *Subgroup meta-analysis of zebrafish ROS by stressor/model*

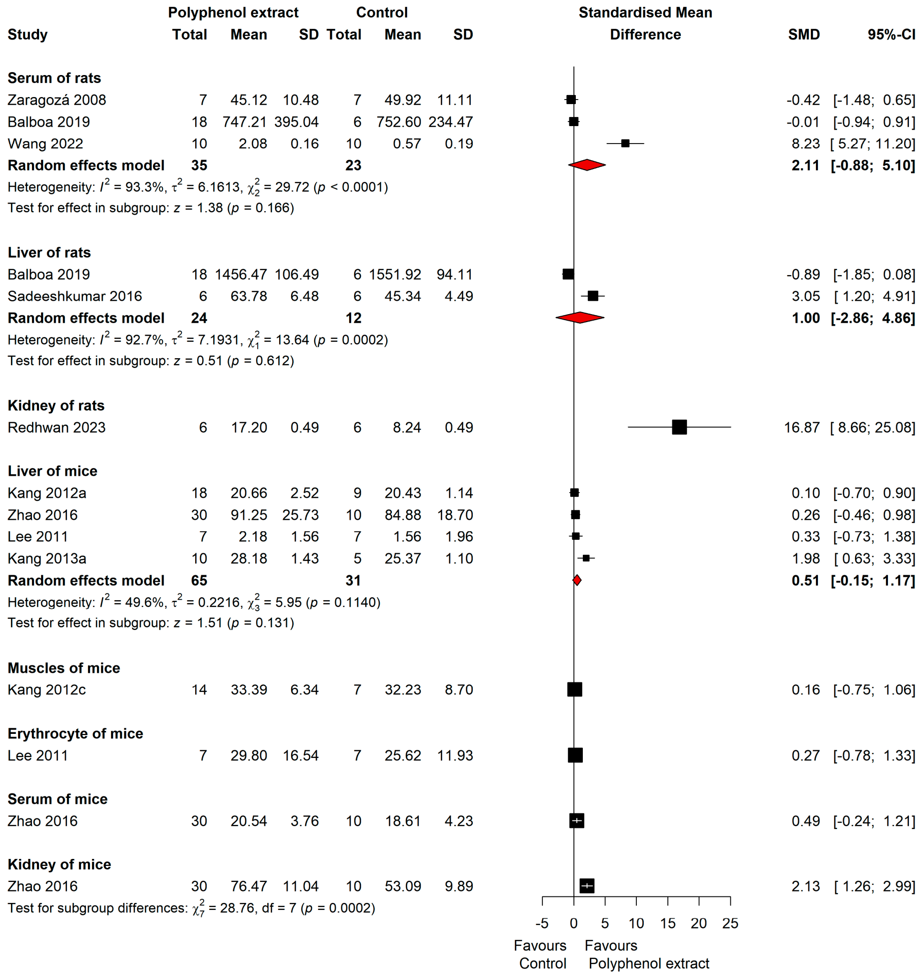

Supplementary Figure S4. *Species-stratified (rats vs mice) subgroup analysis of rodent CAT activity by sample type*

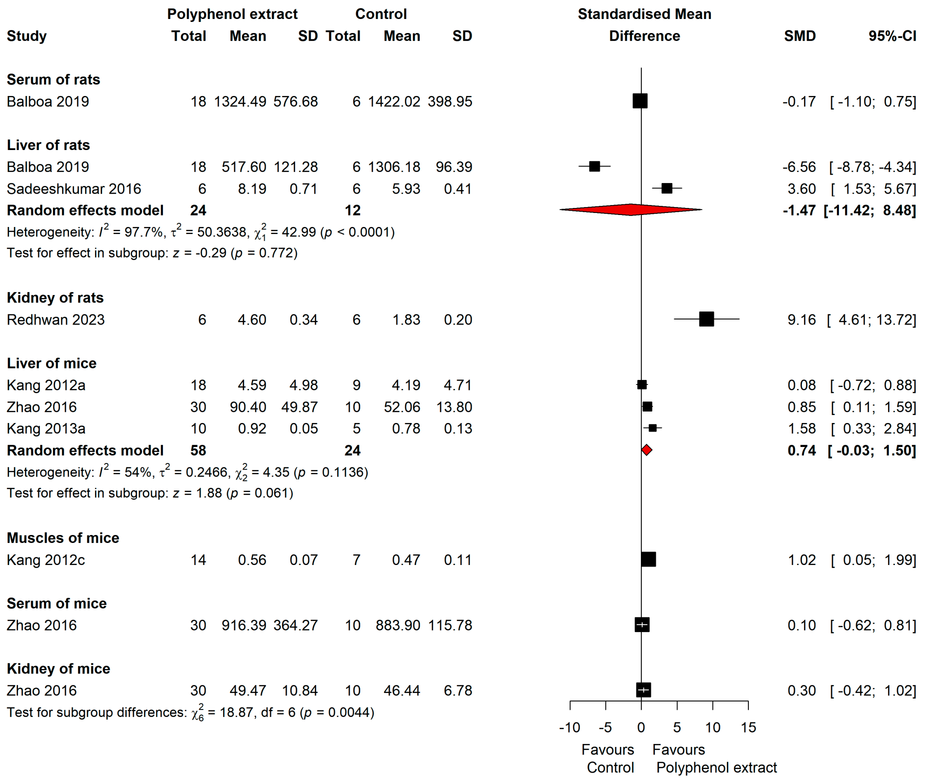

Supplementary Figure S5. *Species-stratified (rats vs mice) subgroup analysis of rodent GPx activity by sample type*

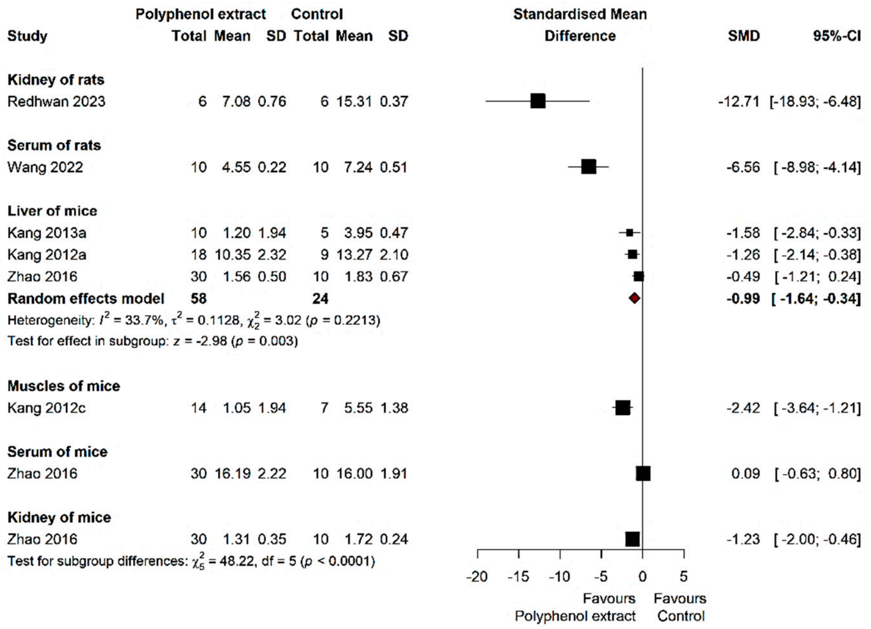

Supplementary Figure S6. *Species-stratified (rats vs mice) subgroup analysis of rodent MDA levels by sample type*

Study	Polyphenol extract Total	Mean	SD	Control Total	Mean	SD	SMD	95%-CI
**Serum of rats**								
Balboa 2019	18	12.24	6.91	6	11.33	4.95	0.13	[-0.79; 1.06]
Zaragozá 2008	7	690.00	79.35	7	603.75	64.48	1.12	[-0.04; 2.27]
Wang 2022	10	14.22	1.14	10	6.30	0.95	7.23	[4.59; 9.86]
**Random effects model**	**35**			**23**			**2.49**	**[-0.29; 5.26]**
Heterogeneity: I^2 = 92%, τ^2 = 5.3205, χ^2_2 = 24.87 (p < 0.0001)								
Test for effect in subgroup: z = 1.75 (p = 0.080)								
**Liver of rats**								
Balboa 2019	18	34.26	2.87	6	35.09	4.41	-0.24	[-1.17; 0.68]
Sadeeshkumar 2016	6	5.86	0.52	6	3.95	0.45	3.62	[1.54; 5.71]
**Random effects model**	**24**			**12**			**1.57**	**[-2.21; 5.36]**
Heterogeneity: I^2 = 91%, τ^2 = 6.8071, χ^2_1 = 11.08 (p = 0.0009)								
Test for effect in subgroup: z = 0.81 (p = 0.415)								
**Liver of mice**								
Zhao 2016	30	92.47	22.64	10	94.50	17.86	-0.09	[-0.81; 0.62]
Kang 2012a	18	118.37	11.25	9	109.91	19.53	0.57	[-0.25; 1.39]
Lee 2011	7	30.83	6.43	7	23.91	6.64	0.99	[-0.14; 2.12]
Kang 2013a	10	47.46	0.73	5	46.22	1.12	1.34	[0.13; 2.55]
**Random effects model**	**65**			**31**			**0.57**	**[-0.04; 1.19]**
Heterogeneity: I^2 = 42.7%, τ^2 = 0.1683, χ^2_3 = 5.24 (p = 0.1550)								
Test for effect in subgroup: z = 1.82 (p = 0.068)								
**Muscles of mice**								
Kang 2012c	14	41.73	12.22	7	34.15	11.85	0.60	[-0.33; 1.53]
**Erythrocyte of mice**								
Lee 2011	7	22.08	6.64	7	15.06	2.78	1.29	[0.10; 2.48]
**Serum of mice**								
Zhao 2016	30	96.98	12.33	10	78.61	13.62	1.42	[0.64; 2.21]
**Kidney of mice**								
Zhao 2016	30	69.13	8.29	10	87.27	3.84	-2.38	[-3.28; -1.48]
Test for subgroup differences: χ^2_6 = 48.18, df = 6 (p < 0.0001)								

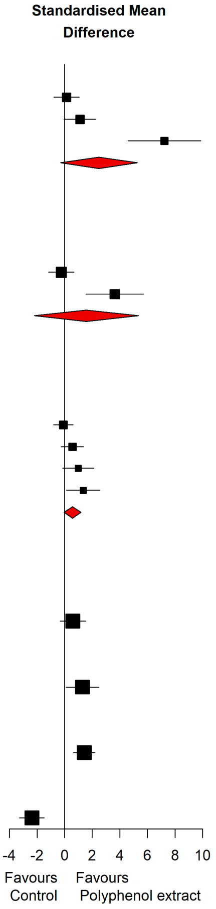

Supplementary Figure S7. *Species-stratified (rats vs mice) subgroup analysis of rodent SOD activity by sample type*
